# Flip Angle Errors in Actual Flip Angle Imaging Using Polyvinylpyrrolidone/Water‐Based Phantoms

**DOI:** 10.1002/mrm.70136

**Published:** 2025-10-18

**Authors:** Niklas Himburg, Max Lutz, Lorenz Mitschang, Jan Gregor Frintz, Sebastian Schmitter

**Affiliations:** ^1^ Physikalisch‐Technische Bundesanstalt (PTB) Berlin Germany; ^2^ Technische Universität Berlin Institut für Physik Und Astronomie Berlin Germany; ^3^ Center for Magnetic Resonance Research University of Minnesota Minneapolis Minnesota USA; ^4^ Medical Physics in Radiology German Cancer Research Center (DKFZ) Heidelberg Germany

**Keywords:** actual flip angle imaging, AFI, $B_{1}^{+}$ mapping, PVP, RF spoiling, ultrahigh field MRI

## Abstract

**Purpose:**

The aim of this work is to report and investigate unexpected errors in actual flip angle imaging (AFI) applied to phantom fillings based on polyvinylpyrrolidone (PVP).

**Methods:**

Flip angle maps of PVP/water mixtures with different PVP ratios were acquired at 3 T and 7 T using a 3D cartesian AFI. The influence of spoiling was investigated by varying the RF‐spoiling phase increment *Φ*_0_ for different settings of the spoiling gradient moments. The results were compared with a reference method and EPG simulations. AFI measurements with varying *TE* were carried out to investigate non‐spoiling‐related errors. Furthermore, ^1^H‐NMR spectroscopy was performed on PVP samples.

**Results:**

Measurements reveal spoiling curves of PVP/water mixtures that lack the symmetry around *Φ*_0_ = 90° that has been observed in prior work. Depending on the *TE* and PVP ratio, substantial deviations from the reference value are found, which are not observed in water samples. Varying the *TE* shows oscillatory behavior of the AFI signals in PVP/water mixtures that match with the existence of PVP bands found in ^1^H‐NMR spectra. Including a signal component with a precession frequency that matches a PVP band into the simulations allows the reproduction of the asymmetric spoiling curves.

**Conclusion:**

Under certain conditions, AFI of PVP/water mixtures suffers from errors originating from interferences of the water and PVP signal components. This can severely reduce the accuracy of the AFI and must be considered for $B_{1}^{+}$ mapping with AFI. The impact of this effect can be reduced by increased *TE* as well as high PVP ratios.

## 1 Introduction

MRI at ultra‐high fields (UHF, ≥7 T) allows higher image resolution and acquisition speed compared with clinical field strengths due to an increased SNR [1]. However, UHF‐imaging suffers from inhomogeneous $B_{1}^{+}$ fields which lead to spatially varying flip‐angle (FA) distributions [1]. This alters the signal strength and thus contrast of the image and can even lead to signal voids (“drop‐outs”).

FA maps are essential for a number of applications like the correction of *T*_1_ maps [2, 3, 4], Electrical Properties Tomography (EPT) [5], transmit‐sensitivity mapping [6] and validation measurements of UHF transmit coils [7, 8]. A fast, robust, and 3D imaging technique used to measure the inhomogeneous flip angle distribution is actual flip angle imaging (AFI) [9]. Since it is a 3D acquisition, it achieves high SNR levels that allow for higher resolution, if needed.

Many of the FA maps applications, especially coil validation measurements, are frequently performed in phantoms. For such uses, the phantom's dielectric properties should be comparable to those of human tissues, which ensures the formation of an RF field pattern inside the phantom similar to the in vivo case [10]. Mixing polyvinylpyrrolidone (PVP) and water presents a solution to achieve tissue‐equivalent relative permittivity values (*ε*_*r*_), while the (electrical) conductivity of the sample can be easily adjusted by adding NaCl [11]. PVP allows achieving *ε*_*r*_ values down to 40 [11], is a non‐toxic substance [12] and is simple to store and to handle. Due to these properties, solutions of PVP and water have been used as phantom fillings for general quality assurance [13, 14] and coil validation/SAR measurements [15, 16, 17, 18, 19, 20]. Considering the advantages of PVP as a phantom material and the AFI as the mapping technique, the combination of both presents a suitable approach for a number of MRI applications, especially at, but not limited to [5, 20], UHF [7, 8].

Applications like coil validation measurements require accurate mapping of the flip angle. Prior work [21, 22] showed that the accuracy of the AFI deteriorates under poor gradient spoiling conditions and depends on the choice of the RF‐spoiling phase increment Φ_0_. In our own measurements using water phantoms, we could reproduce and confirm such reports. However, when we replaced the water phantom with a PVP‐water phantom with matched *T*_1_ value and applied the identical AFI acquisition with the same spoiling parameters, we measured reduced FA values. Such findings triggered an in‐depth investigation, whose results will be presented here.

In this work, we report deviations of the measured FAs from reference values that exceed the expected spoiling‐related errors in PVP‐based phantom fillings. The resulting inaccuracies in AFI‐FA mapping are quantified under different spoiling conditions at 3 T and 7 T, and are compared with results obtained with water phantoms with matched *T*_1_ values. Furthermore, the origin of these deviations is investigated by measurements of the *TE* dependency of AFI and ^1^H‐NMR spectroscopy of different PVP solutions, as well as extended phase graph (EPG [23]) simulations. The goal of this work is to explain the underlying error mechanism and provide recommendations on how the measurement deviations can be reduced for accurate quantifications of FA distributions in PVP‐based phantoms using AFI.

## 2 Methods

### 2.1 Phantom Preparation

The phantom used for the imaging experiments consists of 12 plastic tubes of 17 cm length and 4 cm diameter (cf. Table 1). Four of them were filled respectively with 23, 38, 44, and 50 percentage by weight (wt.%) PVP (Sokalan K30P, BASF SE, Ludwigshafen, Germany) and distilled water, termed PVP5–PVP8 in the following. The PVP ratios are chosen such that the *ε*_*r*_ values cover a range of values (66.4, 57.8, 53.3, 50.1) to be found in human tissue [11]. Tissue‐like conductivity values (*σ*) of 0.62, 0.60, 0.55, and 0.56 S/m were achieved by adding NaCl. A preservative (Germaben II, ISP Sutton Laboratories) was used in a concentration of 1 wt% to prevent microbial growth in the PVP solutions. Permittivity and conductivity values were measured using an impedance analyzer (Agilent, 4396B) after a waiting period of 1–2 days after mixing to ensure the absence of air bubbles. To further investigate the influence of the PVP/water ratio, four additional tubes termed PVP1–PVP4 were filled with PVP/water solutions in the range of 5–20 wt% PVP yielding *ε*_*r*_ values of 76.4, 73.6, 70.6, and 67.5, which are higher than those observed in muscle (58.2) and white matter (43.8) [11].

**TABLE 1:** Overview of the PVP ratios and dielectric properties (*ε*_*r*_, *σ*) of the PVP phantoms used for the imaging experiments.

Tube	PVP ratio (wt%)	Contrast agent (vol%)	*ε*_*r*_	*σ* (S/m)	*T*_1_ at 3 T (ms)	*T*_1_ at 7 T (ms)	*T*_2_ at 3 T (ms)	*T*_2_ at 7 T (ms)	ADC (μm^2^/s)
PVP1	5	0	76.4	0.57	2347	2442	1195	1028	1870
PVP2	10	0	73.6	0.61	2037	2106	1112	898	1610
PVP3	15	0	70.6	0.59	1731	1838	962	739	1380
PVP4	20	0	67.5	0.51	1501	1618	790	603	1200
PVP5	23	0	66.4	0.62	1292	1401	780	604	990
PVP6	38	0	57.8	0.60	751	808	475	327	530
PVP7	44	0	53.3	0.55	546	609	343	231	350
PVP8	50	0	50.1	0.56	410	486	250	179	240
W1	0	0.016			1301	1291	585	380	1930
W2	0	0.038			815	808	525	390	1980
W3	0	0.060			587	583	397	318	1950
W4	0	0.084			455	452	328	267	1910

*Note: T*_1_, *T*_2_, and *ADC* values were needed for simulation of the AFI in specific phantoms and measured beforehand. *T*_1_ values of PVP5–PVP8 and W1–W4 were matched by doping the water phantoms with contrast agent.

For comparison, four tubes (termed W1–4) were filled only with distilled water and doped with a Gadolinium‐based contrast agent (ProHance, Bracco Imaging Deutschland GmbH). The contrast agent was used to achieve *T*_1_ relaxation values similar to the PVP5–PVP8 tubes at 3 T. *T*_1_ values were confirmed by measurements using a spin‐echo inversion recovery sequence. *T*_2_ values were acquired using a spin‐echo sequence and the *ADC* values by a diffusion‐weighted spin‐echo EPI. Isotropic diffusion was assumed in the PVP solutions. All phantom parameters relevant for the simulation (*T*_1_, *T*_2_, *ADC*), together with their dielectric properties, are listed in Table 1.

### 2.2 Imaging Experiments

A 3D cartesian AFI sequence was applied in all imaging experiments with non‐selective, rectangular‐shaped excitation pulses of 0.5 ms pulse duration. RF‐spoiling was applied combined with spoiling gradients along the read‐out direction. The phase of the *j*th RF‐pulse (Φ_*j*_) was altered according to the following recurrence relation that was proposed for FLASH sequences by Crawley et al. [24] and used in the first implementation of AFI [9]:

(1) $$\Phi_{j}=\Phi_{j}-1+j\Phi_{0}$$

with the RF‐spoiling phase increment Φ_0_. Common parameters for all scans were: *TR*_1_/*TR*_2_ = 25/125 ms (*n* = *TR*_1_/*TR*_2_ = 5), nominal flip angle *α*_nom_ = 60°, FOV = 192 × 192 × 200 mm^3^, resolution = 2 × 2 × 5 mm^3^. To ensure a steady state magnetization, dummy AFI scans were applied for the first 2.0 s of image acquisition.

Images were acquired at 3 T (Magnetom Cima.X, Siemens Healthineers, Erlangen, Germany) using a 64Rx head coil and at 7 T (Magnetom 7 T, Siemens Healthineers, Erlangen, Germany) using a 1Tx/32Rx head coil (Nova Medical, Wilmington, MA).

At 7 T, six tubes (W1–3, PVP1–3) were placed in a cylindrical phantom holder that was filled with water and doped with contrast agent.

**FIGURE 1:**
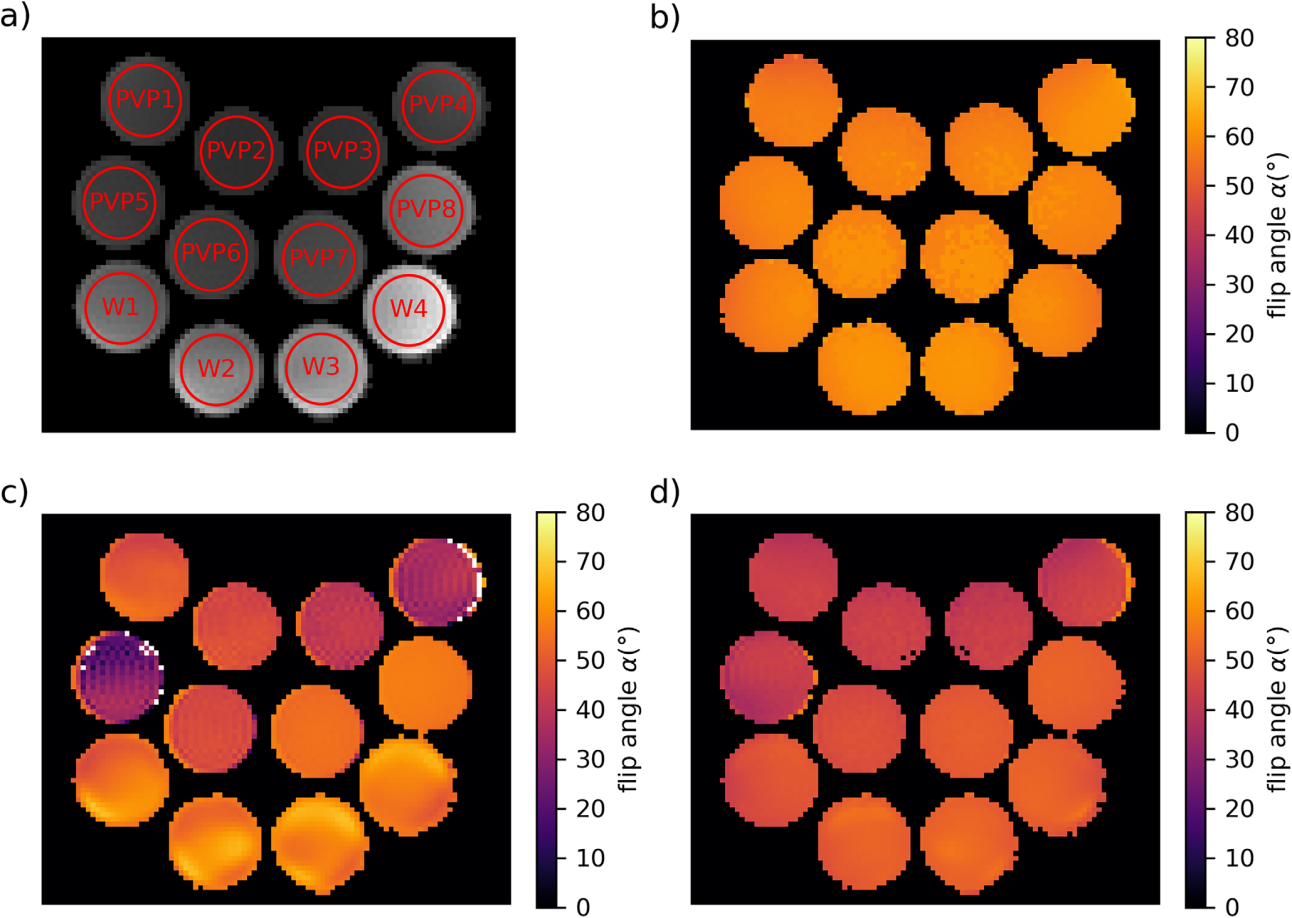
Transverse flip angle maps of the 12 tubes resulting from the imaging experiments at 3 T. The amplitude image (a) is one of the two GRE images used for calculating the AFI map shown in (c). The names of the tubes are indicated in (a) in red. The reference map acquired with PEX is depicted in (b) and shows flip angles deviating less than 3% from *α*_nom_ = 60° in all tubes. The AFI maps shown in (c) and (d) were acquired with *A*_G1_/*A*_G2_ = 117.5/587.5 mT ms/m, *TE* = 1.9 ms, Φ_0_ = 40° (c) and Φ_0_ = 100° (d). In both, an underestimation of the flip angles compared with the reference map can be found in some PVP filled tubes, especially in PVP3, PVP4, PVP5 and PVP6. This underestimation shows a strong dependence on Φ_0_ with, for example, a mean flip angle of *α* = 28.8° in PVP5 in (c) and *α* = 41.1° in (d). Calculation of the flip angle failed in some pixels in (c) which are shown in white.

For analysis of all scans, circular ROIs were manually placed over the tubes (Figure 1a), and the mean and standard deviation inside were evaluated. For one measurement set, all measurements were evaluated with the exact same ROIs. Only the AFI slice at the position of the reference scan was used as the FA varied in slice direction.

#### 2.2.1 Spoiling Curves

To investigate possible spoiling related errors, AFI scans were applied to the phantom containing all 12 tubes with varying RF‐spoiling phase increments in the range of Φ_0_ = 0°–180° in steps of 20°. At 3 T, three sets (I–III) of these “spoiling curves” were acquired with different spoiling gradient moments: (I) *A*_G1_/*A*_G2_ = 117.5/587.5 mT ms/m, (II) *A*_G1_/*A*_G2_ = 234.9/1174.5 mT ms/m, and (III) *A*_G1_/*A*_G2_ = 469.7/2348.5 mT ms/m. These values translate into a 20π, 40π, and 80π dephasing over a 2.0 mm voxel. *TE* was fixed to *TE* = 1.9 ms for all spoiling curve AFI acquisitions at 3 T.

Another three sets of spoiling curves were acquired at 7 T. Here, the spoiling gradient moment I was constantly applied (*A*_G1_/*A*_G2_ = 117.4/587.0 mT ms/m) while different *TE* values of *TE* = 1.9 ms, *TE* = 2.5 ms, and *TE* = 3.0 ms were applied.

#### 2.2.2 TE Dependence

The echo time dependency of the non‐spoiling related errors was investigated by acquiring AFI scans with different *TE* values. Values range from *TE* = 1.5 ms to *TE* = 6.1 ms in steps of 0.2 ms. Two of these measurements were taken at 3 T, the first again with intermediate gradient spoiling moments I (*A*_G1_/*A*_G2_ = 117.4/587.0 mT ms/m) and the second acquisition with maximal applicable gradient moments (IV) of *A*_G1_/*A*_G2_ = 705/3525 mT ms/m. At 7 T, the *TE* dependency was measured only with spoiling moment I. In all these measurements, Φ_0_ was set to 120°, which is close to the value of Φ_0_ = 117° that was used in the first publication of the AFI method [9] and close to the value proposed by Nehrke [21] (Φ_0_ = 129.3°).

#### 2.2.3 Reference Method

To evaluate the strength of measurement errors when using the AFI sequence, the FA was measured by a reference method before a group of AFI scans. A slow but accurate 2D‐preparation‐based sequence [25] (PEX) was used.

Here, a rectangular preparation pulse with a given nominal FA and a duration of 0.5 ms was applied. After the preparation pulse, the transverse magnetization was destroyed by gradient spoiling (gradient moment 117.5 mT ms/m) followed by the Cartesian read‐out gradient for a single k‐space line. To reduce the influence of residual signals, long repetition times of 7000 ms were used. Ten images were acquired where at 3 T the nominal FA of the preparation pulse ranged from 0° to 135° in steps of 15° (reference voltage: 256 V), and the resolution matched the AFI (2 × 2 × 5 mm^3^). This led to an acquisition time of 1 h and 26 min. At 7 T, the transmission voltage was varied directly from 0 to 270 V in steps of 30 V (reference voltage: 100 V). A detailed explanation of the method was provided in the study of Lutz et al. [26].

### 2.3 Spectroscopy Experiments

Two samples containing PVP at 20 and 50 wt%, respectively, in deuterium oxide (purity ≥ 99.8%) were prepared for analysis by ^1^H‐NMR spectroscopy on a benchtop spectrometer at field strength 1.88 T (“Fourier‐80” Bruker BioSpin, Ettlingen, Germany). No preservative was added to these samples. Standard spectra of 128 accumulations were acquired at a sample temperature of 298 K using a 90° excitation pulse of length 15.25 μs.

The *T*_1_ of ^1^H assigned to PVP and residual water, respectively, was determined by inversion recovery. For the sample containing PVP at 20 wt% the recovery delay was set in the range 0–200 ms (step size 10 ms) for PVP and 0–14 s (step size 2 s) for water signals, respectively, and similarly for the sample with PVP at 50 wt% in the range again 0–200 ms (step size 10 ms) and 0–1 s (step size 50 ms), respectively. The recovery delay at vanishing signal intensity, *t*_0_, was used to calculate $T_{1}=t_{0}/\ln2$.

### 2.4 Simulations

Simulation of the spoiling curves was done by 1‐dimensional extended phase graph simulations (EPG) [23]. The simulation is based on publicly available code [27] and was implemented in Python (version 3.12.2). It was extended to simulate RF‐ and gradient‐spoiled AFI sequences and to include isotropic diffusion [23]. No imaging gradients (readout and phase‐encoding) were simulated in favor of reduced computational cost, as their gradient moments are > 10 times smaller than the smallest spoiler gradient moment. The gradient moments were discretized to multiples of 2*π* dephasings over one slice in the z‐direction and restricted the choice of spoiler gradient moments in the imaging experiments to multiples of 11.75 mT ms/m.

The flip angles were calculated according to Yarnykh [9]:

(2) $$\alpha =\arccos\left(\frac{\mathrm{rn}-1}{n-r}\right),r=\frac{|S_{2}|}{\left|S_{1}\right|},n=\frac{TR_{2}}{TR_{1}}$$

with simulated complex signals during the first and second repetition time *S*_1_ and *S*_2_.

To incorporate interference effects of different resonance frequencies into the simulation, two EPG simulations were performed for one AFI acquisition. For the first one, resonant excitation was assumed and *T*_1_, *T*_2_, and *ADC* parameters were set according to Table 1 to simulate signal strengths *S*_1,res_ and *S*_2,res_. The second simulation with the same ADC values was assumed to be off‐resonant by Δ*ν* with resulting signals *S*_1,off_ and *S*_2,off_. Simulation parameters were set to resemble off‐resonant signal components appearing in the ^1^H‐NMR spectra (Δ*ν* = 320 Hz/747 Hz at 3 T/7 T). Off‐resonant excitation effects were included by using an extended EPG‐excitation matrix that was derived using the method shown by Weigel [23]. Combination of the complex resonant and off‐resonant signal components was done according to:

(3) $$S_{i}=S_{i,\mathrm{res}}\left(T1_{\mathrm{res}},T2_{\mathrm{res}},\mathrm{ADC}\right)+w\cdot S_{i,\mathrm{off}}\left(T1_{\mathrm{off}},T2_{\mathrm{off}},\mathrm{ADC},\Delta \nu \right)e^{i\left(2\pi \cdot \Delta \nu \cdot \mathrm{TE}+\varphi \right)}$$

with *i* = 1,2, and accounts for the phase accumulated by *S*_*i*,off_ in the rotating frame during *TE*. *w* and *φ* were used as fitting parameters to match simulated and measured spoiling curves. Note, that *φ* is independent of the spoiling increment Φ_0_. Parameters were fitted by minimizing the root‐mean‐square deviation between the simulated and measured spoiling curves.

## 3 Results

Figure 1 showcases exemplary results of the imaging experiments performed at 3 T, which is similar to the experiment mentioned in the introduction that led to this investigation. Figure 1a shows the AFI magnitude image acquired after *TR*_1_ along with the tube annotation and ROIs in a transverse, central slice (20 of 40) through the 12 tubes (cf. Table 1). Here the top row contains PVP concentrations with *ε*_*r*_ between 67.5 and 76.4, the middle row contains PVP tubes with *ε*_*r*_ values found in human tissues (66.4, 57.8, 53.3, 50.1), and the lower row the matching water‐filled tubes. As can be seen, PVP vials exhibit a lower signal intensity due to the lower water content. The result of the 2D‐PEX scan is presented in Figure 1b. Here, as expected, the mean flip angles in all tubes are close to the nominal flip angle *α*_nom_ = 60° with deviations below 3%.

**FIGURE 2:**
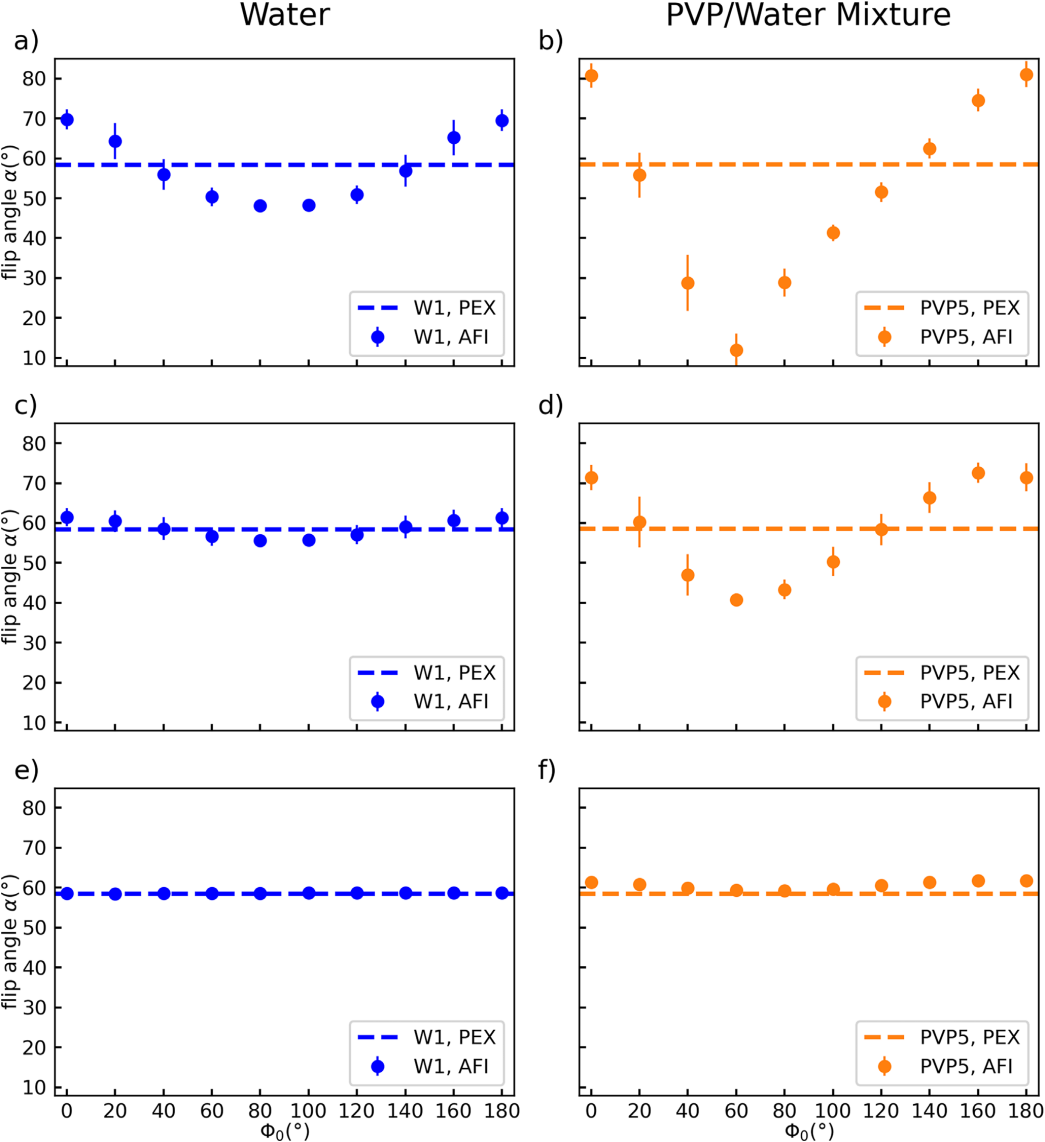
Spoiling curves of tube W1 and PVP5 acquired at 3 T with *TE* = 1.9 ms and spoiling gradient moments of *A*_G1_/*A*_G2_ = 117.5/587.5 mT ms/m (a + b), *A*_G1_/*A*_G2_ = 234.9/1174.5 mT ms/m (c + d), and *A*_G1_/*A*_G2_ = 469.7/2348.5 mT ms/m (e + f). The reference value for each measurement is indicated by a dashed line. AFI results show increased deviations from the reference value in PVP5 compared with W1 at some Φ_0_ values with a maximum deviation of −79% at Φ_0_ = 60° (b). In contrast to the water curves, the spoiling curves of PVP are not symmetric around Φ_0_ = 90°. These effects are reduced for increased spoiling gradient moments. All subfigures share the *x*‐ and *y*‐axis and error bars indicate the standard deviation in the tube.

This is different in the AFI maps shown in Figure 1c,d. Both belong to the first set of spoiling curves with *A*_G1_/*A*_G2_ = 117.5/587.5 mT ms/m and *TE* = 1.9 ms. In Figure 1c, Φ_0_ was set to Φ_0_ = 40°. Here, the measured FAs differ between the PVP tubes, with the lowest mean flip angle of *α* = 28.8° found in PVP5 (23% PVP ratio). For maximum and minimum PVP ratios, the flip angle gradually increases to *α* = 56.7° in PVP8 (50% PVP ratio) and *α* = 50.0° in PVP1 (5% PVP ratio). The white pixels found in tubes PVP4 and PVP5 indicate that flip angle calculation by Equation (2) failed; that is, the argument of the arccos function was outside its definition range [−1, 1]. The water tubes with matched *T*_1_ times (W1–4) show only small differences between each other, with mean flip angles ranging from 55.5° to 59.5°. The phenomenon of the PVP ratio dependent flip angles is qualitatively lower in Figure 1d with Φ_0_ = 100° but still visible. A quantitative description of this effect is given in the form of spoiling curves in Figure 2.

### 3.1 Dependence on RF and Gradient Spoiling

The spoiling curves of tubes PVP5 and W1 with matched *T*_1_ times acquired at 3 T are shown in Figure 2 for spoiling gradient moments of *A*_G1_/*A*_G2_ = 117.5/587.5 mT ms/m (Figure 2a,b), *A*_G1_/*A*_G2_ = 234.9/1174.5 mT ms/m (Figure 2c,d), and *A*_G1_/*A*_G2_ = 469.7/2348.5 mT ms/m (Figure 2e,f). Each datapoint reflects the mean FA measured inside a circular region of interest that was placed inside each tube in the central slice of the AFI maps (e.g., Figure 1c,d).

These plots reveal asymmetric spoiling curves for PVP while the spoiling curves for water are symmetric with an orthogonal symmetry line at Φ_0_ = 90°. For both tubes presented in Figure 2, the dependency of the measured flip angle on Φ_0_ decreases with increasing gradient spoiling moment. At the highest gradient moment (Figure 2e,f), the results of tube W1 reflect no visible dependency on Φ_0_ and mean deviation between AFI and PEX amounts to maximal 0.6%. This regime was defined by Yarnykh [22] as “complete spoiling.”

With decreasing gradient spoiling moment, both curves illustrate an increased Φ_0_‐dependent deviation from the PEX results. For PVP5 the minimum in the spoiling curves is found at Φ_0_ = 60° for spoiling moment I and II, where the deviation from the reference value amounts to −79% (Figure 2b) for moment I. For comparison, the deviations from the reference value in W1 range from −13% to +20% for moment I.

To quantify the asymmetry of the PVP spoiling curves with respect to Φ_0_ = 90°, we use the difference in FA between the two points where the minima of the curve are observed ($\Delta \alpha =\alpha \left(\Phi_{0}=120^{{}^{\circ}}\right)-\alpha \left(\Phi_{0}=60^{{}^{\circ}}\right)$) that appear in PVP under certain parameter settings. As these two points are equidistant to the symmetry line at Φ_0_ = 90° found in water, a perfectly symmetric spoiling curve has Δ*α* = 0. For PVP5 the asymmetry amounts to Δ*α* = 39.3° in (b), Δ*α* = 17.9° in (d), and Δ*α* = 1.3° in (f). In contrast, the spoiling curves of the W1 tube show a high degree of symmetry with |Δ*α*| < 0.5° for all three gradient spoiling moments.

The loss of symmetry in the PVP spoiling curves, increased deviations from the reference value, and reduction of these effects for increased spoiling gradient moments were observed in all tubes, but with varying intensity (shown in Figures S1–S5).

**FIGURE 3:**
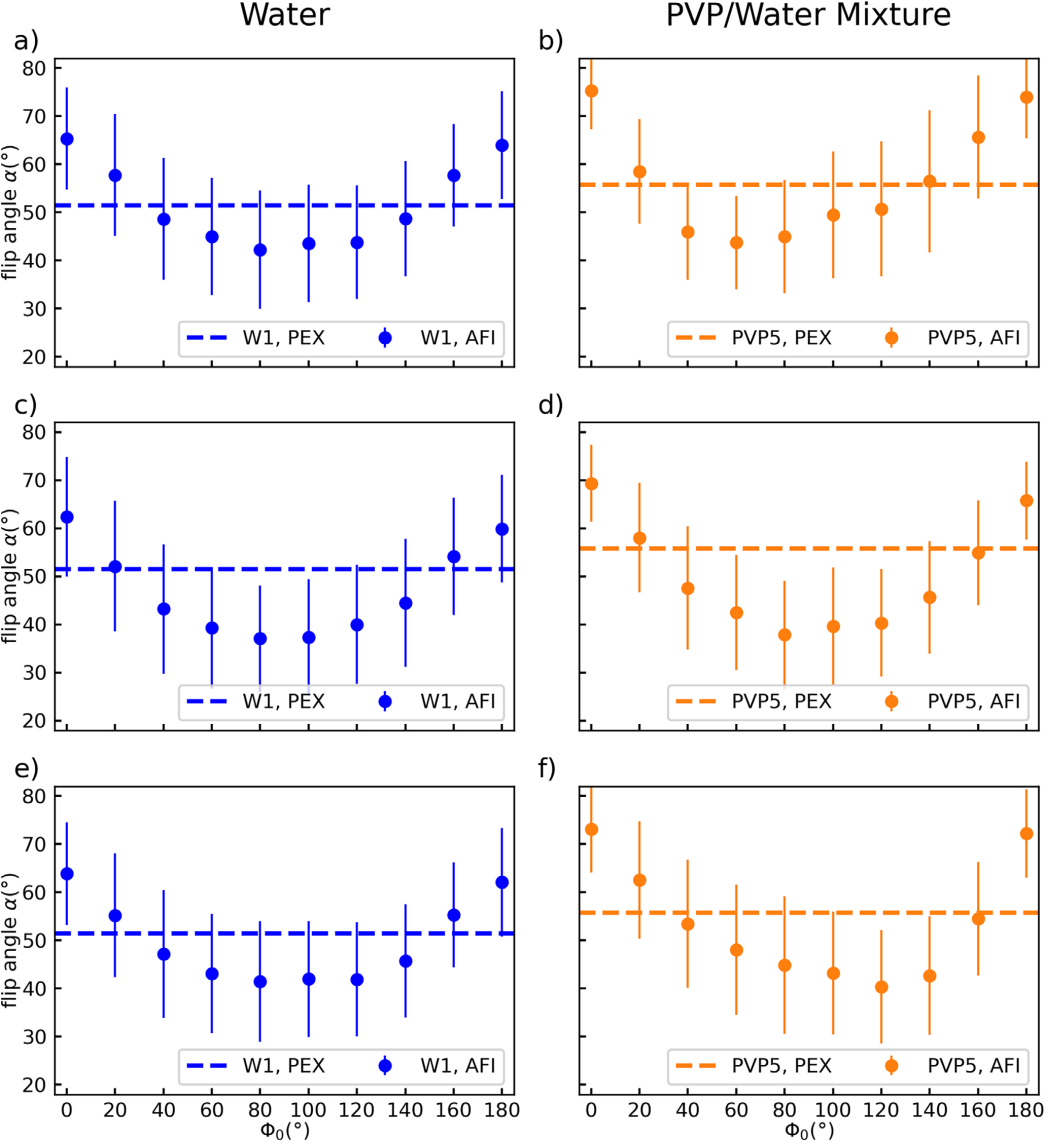
Spoiling curves of tube W1 and PVP5 acquired at 7 T with fixed spoiling gradient moments of *A*_G1_/*A*_G2_ = 117.4/587.0 mT ms/m and echo times of *TE* = 1.9 ms (a + b), *TE* = 2.5 ms (c + d), and *TE* = 3.0 ms (e + f). The reference value for each measurement is indicated by a dashed line, and the axes are shared by all subfigures. Spoiling curves measured in water are symmetric around Φ_0_ = 90° (|Δ*α*| < 1°) and are not changed by the choice of TE. The PVP spoiling curves are asymmetric depending on the *TE* value, with asymmetry values of Δ*α* = 7.2° (b), Δ*α* = −2.3° (d), and Δ*α* = −7.2° (f). Negative asymmetry values indicate a shift of the spoiling curves' minimum from Φ_0_ = 60° to Φ_0_ = 120°.

Figure 3 shows the spoiling curves for tubes W1 and PVP1 acquired at 7 T with different *TE* values of 1.9 ms (Figure 3a,b), 2.5 ms (Figure 3c,d), and 3.0 ms (Figure 3e,f) and gradient moment I. Similar to the results found at 3 T, the spoiling curves of the water tube did not exhibit an asymmetric shape (|Δ*α*| < 1°). Furthermore, all three water curves show similar deviations from the reference value with deviations ranging from (a) −18.4% to 26.4%, (c) −28.5% to 20.4%, and (e) −20.0% to 23.5%. Thus, they generally appear to be not affected by changes of the *TE* value.

In contrast, the spoiling curves obtained in PVP5 also show asymmetric shapes; however, the exact shape changes with the *TE* value. For *TE* = 1.9 ms, the curve's minimum can be found at Φ_0_ = 60° (Δ*α* = 7.2°) as in Figure 2, where the same *TE* was applied. For longer *TE*, however, the minimum is found at Φ_0_ = 120° with an asymmetry of Δ*α* = −2.3° at *TE* = 2.5 ms (Figure 3d) and further increased asymmetry of Δ*α* = −7.2° at *TE* = 3.0 ms (Figure 3f). In contrast to the results at 3 T (Figure 2), the PVP curves show deviations from the reference FA comparable to the ones found in water, ranging from (b) −21.9% to 34%, (d) −32.2% to 24.0%, and (f) −28.1% to 30.5%.

### 3.2 Echo Time Dependence

**FIGURE 4:**
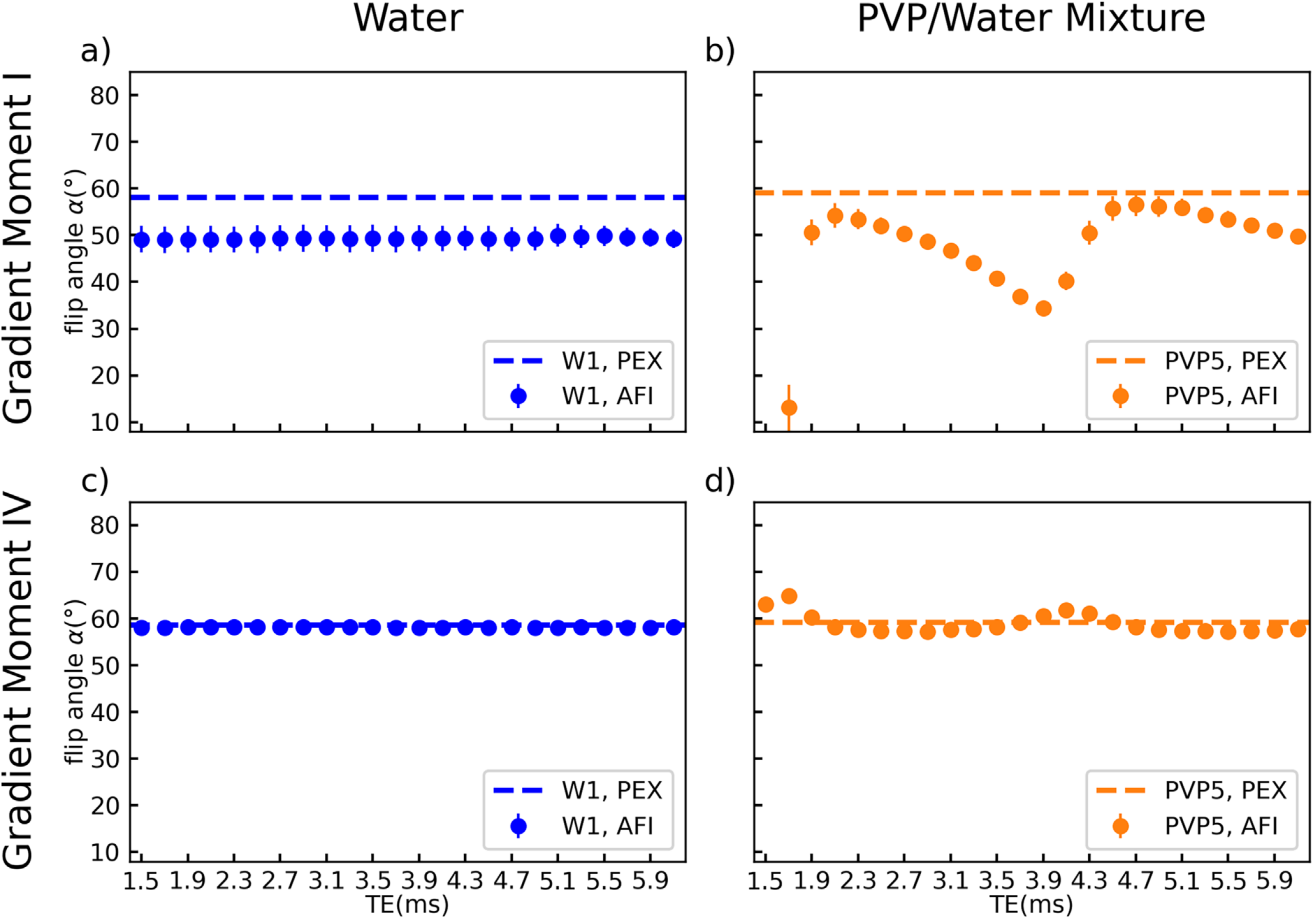
Echo time dependency of the AFI results in tube W1 (a, c) and PVP5 (b, d) measured at 3 T. Φ_0_ was fixed to 120° and the gradient spoiling moments were set to *A*_G1_/*A*_G2_ = 117.4/587.0 mT ms/m (a + b) and *A*_G1_/*A*_G2_ = 705/3525 mT ms/m (c + d). AFI results in the water tube are constant for all *TE* values and show an underestimation of the reference value (represented by a dashed line) for low gradient spoiling moments (a). AFI results in PVP1 show a periodic dependency on the echo time with a period length of 2.4 ms and deviations from the reference value of up to −78% in (b) and + 9.5% in (d). The axes are shared by all subfigures and error bars indicate the standard deviation over the tubes cross section.

**FIGURE 5:**
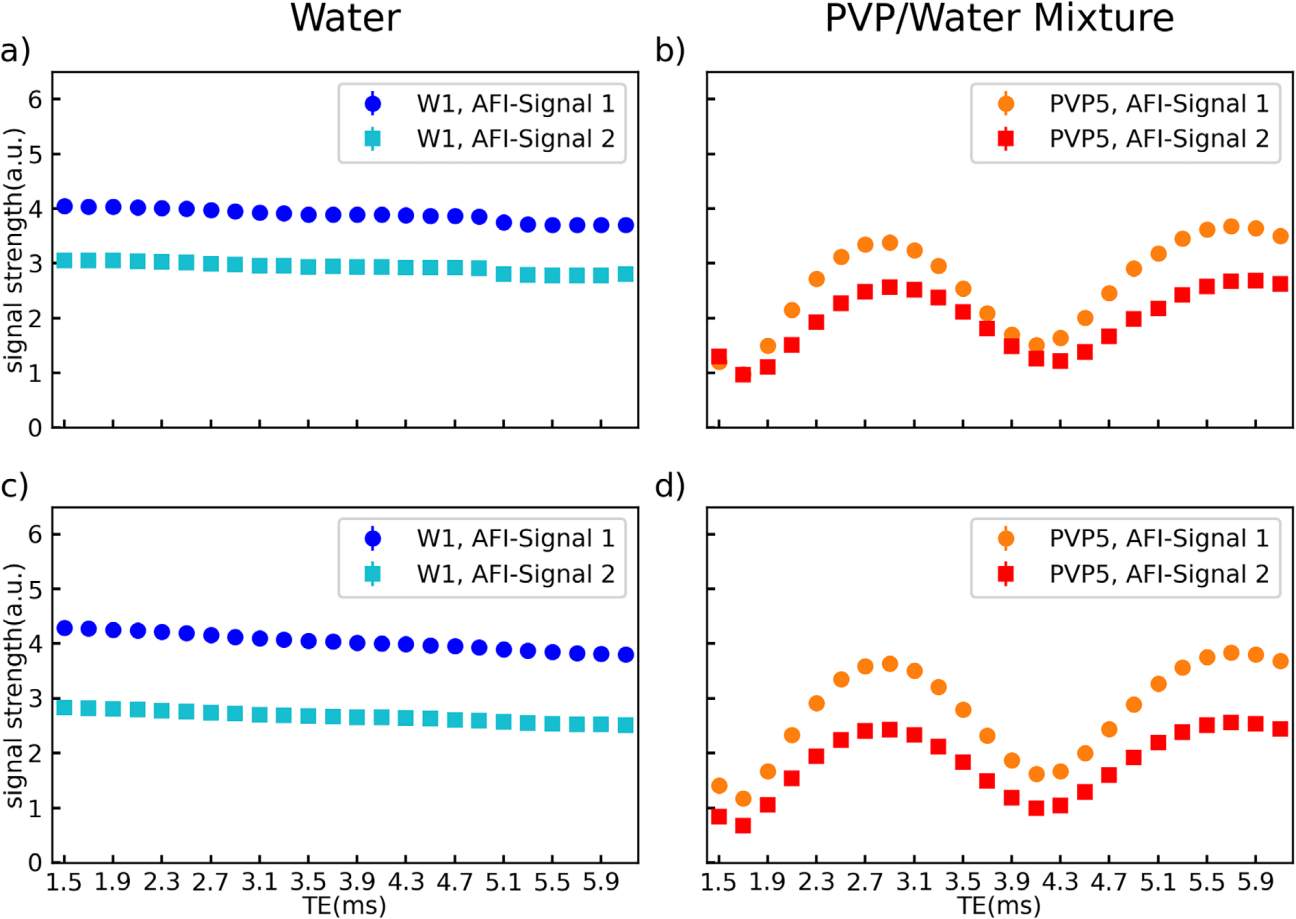
Echo time dependency of the signal strength in the GRE images that were used to calculate the flip angle in tube W1 (a + c) and PVP5 (b + d) shown in Figure 4. Images were acquired at 3 T with Φ_0_ fixed to 120° and gradient spoiling moments set to *A*_G1_/*A*_G2_ = 117.4/587.0 mT ms/m (a + b) and *A*_G1_/*A*_G2_ = 705/3525 mT ms/m (c + d). The signal strength measured in the water tube shows a linear dependence on *TE* while a sine‐like dependency is found for the PVP tube. The sine curves are shifted with respect to each other by 0.2 ms for low gradient spoiling moments (b). The axes are shared by all subfigures and error bars indicate the standard deviation over the tubes cross section.

**FIGURE 6:**
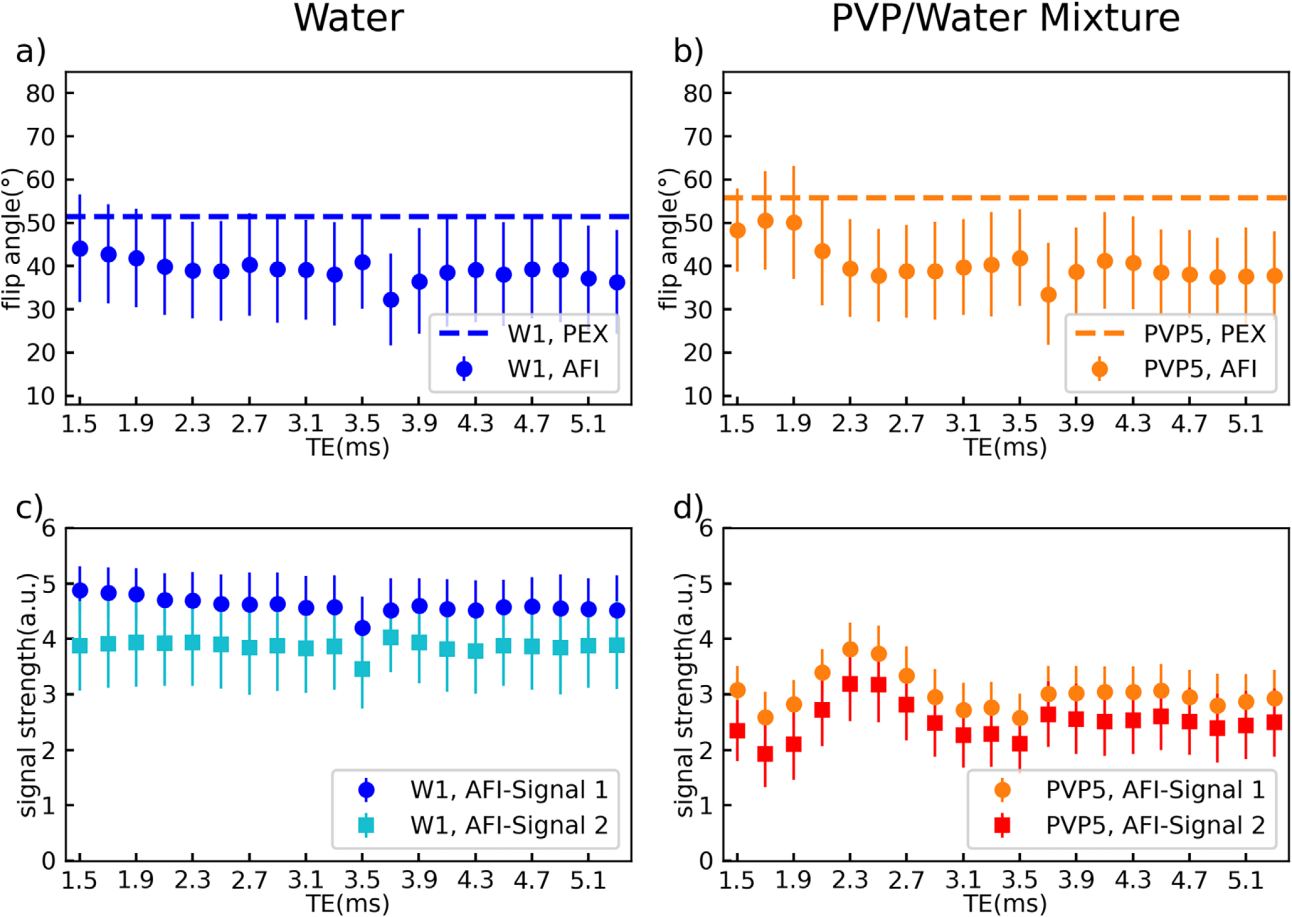
Echo time dependency of the flip angle (a + b) and the signal strength in the GRE images (c + d) acquired in tubes W1 (a + c) and PVP5 (b + d) at 7 T. Φ_0_ was fixed to 120° and gradient spoiling moments set to *A*_G1_/*A*_G2_ = 117.4/587.0 mT ms/m. Reference flip angles are indicated by the dashed lines, and error bars show the standard deviation over the tubes' cross section. The *TE* dependency in the water tube is comparable to 3 T, with linearly decreasing signal strength. Oscillatory behavior of the PVP curves is also present here. However, flip angles (b) show a reduced maximum deviation from the reference value compared with 3 T. Furthermore, the signal strengths (d) show no sine‐like behavior. The drop in signal amplitude found at TEs of 3.5 ms and 3.7 ms is present in all tubes used at 7 T.

The *TE* dependence of the AFI in PVP solutions found in Figure 3 is further investigated in Figures 4, 5, 6. Figure 4 depicts the *TE* dependence of the measured FA in W1 (Figure 4a,c) and PVP5 (Figure 4b,d) at 3 T for spoiling gradient moments of *A*_G1_/*A*_G2_ = 117.4/587.0 mT ms/m (Figure 4a,b) and *A*_G1_/*A*_G2_ = 705/3525 mT ms/m (Figure 4c,d).

The FAs measured in the water tube show a constant underestimation compared with the reference value of −15% for the lower gradient spoiling moment I (Figure 4a) and good agreement with the reference value for the higher gradient spoiling moment IV (Figure 4c). In both plots, however, no dependence of the FA on *TE* can be observed.

In contrast, the PVP curves show a dependency on *TE* that appears to be periodic with a period length of 2.4 ms for both the lower moment (Figure 4b) and the higher moment (Figure 4d). As for the water tube in Figure 4a, the measured FAs for the lower moment in Figure 4b fall below the reference value, with local minima reaching up to −78% at *TE* = 1.7 ms. In contrast, for high gradient spoiling moments (Figure 4d), the curve shows distinct maxima instead of minima, with a maximum deviation of +9.5% at *TE* = 1.7 ms. The first datapoint (at *TE* = 1.5 ms) in subfigure Figure 4b is missing. Here, the calculation of the flip angle failed due to an inversion of the signal amplitude (i.e., *S*_2_ > *S*_1_) that leads to an argument > 1 of the arccos function in Equation (2).

Further results from the same measurement of Figure 4 are provided in Figure 5 showing the signal magnitude of the two GRE images (*S*_1_/*S*_2_) used for FA calculation. For W1 (Figure 5a,c), the signal strength decreases approximately linearly with *TE*, while a drop in signal strength can be observed at *TE* = 5.1 ms (Figure 5a). This drop coincides with a pause in data acquisition of a few hours. Although the differences between the two signals are not constant, their ratio and thus the FA remains the same (Figure 4a,c). In contrast to water, the signals in tube PVP5 (Figure 5b,d) show a sine‐like pattern with a period of 2.4 ms. Here, the differences between the two signals and their ratio change with *TE* and result in the FA variations observed in Figure 4b,d. For the lower gradient spoiling moment I, the two curves are shifted with respect to each other by 0.2 ms (Figure 5b), which exacerbates the resulting FA variations. Here, for *TE* values close to the minima of the curves, the amplitude of *S*_1_ is comparable or even falls below the amplitude of *S*_2_ (*S*_2_ > *S*_1_). Additionally, an increase in signal strength with *TE* can be observed for the PVP curves as the second local maximum of each curve exceeds the first one.

Dependencies of the FA and the individual AFI signals on *TE* are also investigated at 7 T using the same tubes W1 and PVP5 (Figure 6). As for 3 T, the FA measured in W1 (Figure 6a) does not show a clear *TE* dependency but underestimates the reference value by −15% to −30%. The corresponding *TE* dependency on the individual signal magnitudes (Figure 6c) is also comparable with 3 T results (Figure 5a). AFI results for PVP5 (Figure 6b), however, show a maximum deviation of −32% from the reference value at *TE* = 5.1 ms, which is less than half the maximum deviation of −78% found at 3 T (Figure 4b). Similar to 3 T, oscillatory behavior of the FA and signal magnitude curves is visible. However, the *TE* dependency of the signal curves (Figure 6d) appears more complex than the sine‐like shape found at 3 T (Figure 5b,d). The drop in signal amplitude at *TE* = 3.5 ms and *TE* = 3.7 ms is found in all tubes at 7 T.

### 3.3 NMR‐Spectra

**FIGURE 7:**
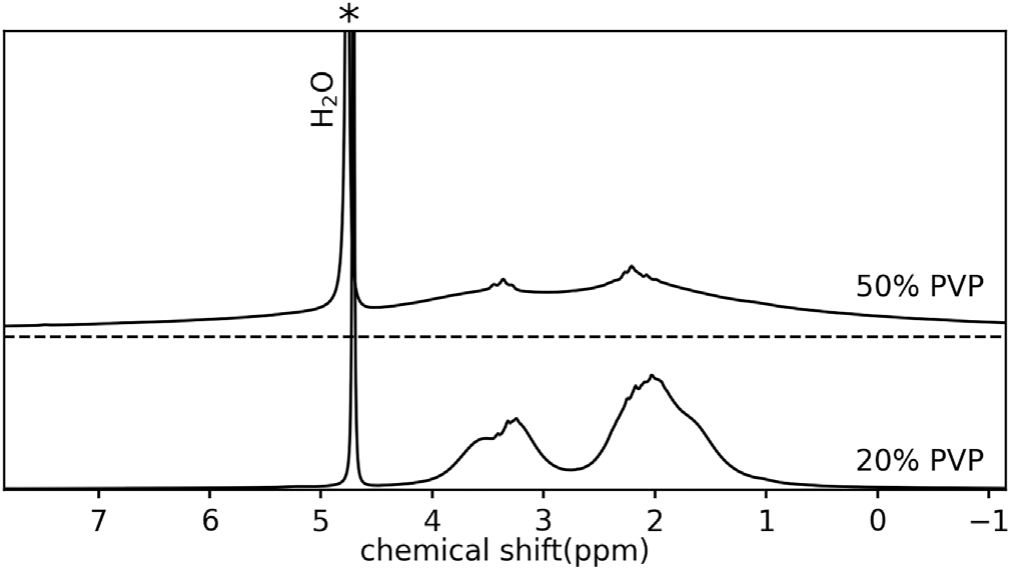
^1^H‐NMR spectra of 20 and 50 wt% PVP in D_2_O. Both spectra contain a signal of residual water at ∼4.7 ppm and two additional bands from PVP located at ∼2.1 and ∼3.3 ppm, respectively. The bands are broadened and reduced in amplitude for 50 wt% PVP in comparison to 20 wt% PVP.

The ^1^H‐NMR standard spectra on a benchtop spectrometer of the two samples with 20 and 50 wt% PVP (in D_2_O), respectively, are shown in Figure 7. A distinct residual water peak can be found that was calibrated to ∼4.7 ppm. In both spectra two additional bands can be recognized. Taking the reduced spectral resolution compared with high resolution spectrometers into account, for 20 wt% PVP the band located at 2.11 ppm can be assigned to side chain methylene protons while the band located at 3.28 ppm is due to main and side chain methylene as well as main chain methine protons of PVP [28]. For 50 wt% PVP in comparison to 20 wt% PVP an overall small signal shift of grossly 0.1 ppm but significant line broadening and amplitude reduction occurs. For example, for the upfield‐shifted band a FWHM of 2.5 ppm at 50 wt% PVP compares to 0.7 ppm at 20 wt% PVP, indicating respective $T_{2}^{*}$ of 1.6 and 5.7 ms. The FWHM of the bands was determined using direct measurement from the spectra.

Interestingly, the PVP assigned bands in either sample experience a common longitudinal relaxation because of an identical recovery delay of vanishing signal intensity in the inversion recovery experiment. The measured *T*_1_ of the PVP/water resonances is 100/4000 ms for 20 wt% and 70/350 ms for 50 wt%, respectively.

### 3.4 EPG‐Simulations

**FIGURE 8:**
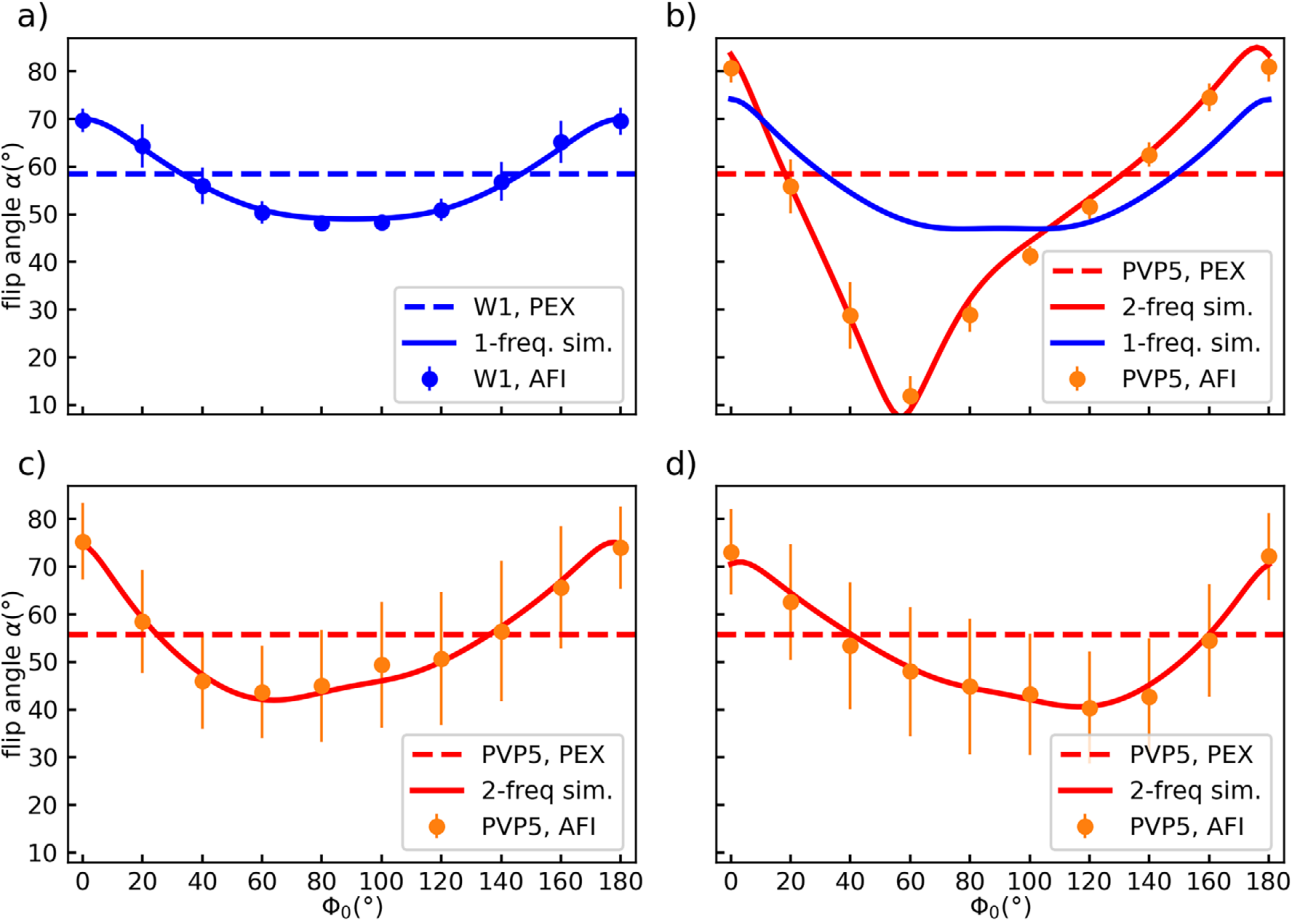
Simulated and measured spoiling curves for (a) W1 and (b) PVP5 at 3 T with *TE* = 1.9 ms as well as (c) PVP6 at 7 T with *TE* = 1.9 ms and (d) PVP6 at 7 T with *TE* = 3.0 ms. All measurements were acquired with gradient spoiling moments of *A*_G1_/*A*_G2_ = 117.4/587.0 mT ms/m. Single‐frequency simulations match with the measured spoiling curves of W1 but do not agree with the asymmetric shape of the PVP curves. Adding an additional precession frequency to the simulation leads to good agreement of simulated and measured PVP spoiling curves including the shift of the curves minimum value to Φ_0_ = 120° in subfigure (d). The reference values used for the spoiling curve simulation are indicated by dashed lines and axes are shared between all subfigures.

Figure 8 presents the EPG simulated and measured spoiling curves for the W1 tube and PVP1 tube at 3 T (Figure 8a,b) as well as those obtained from PVP2 measured at 7 T. The measured spoiling curves alone were already shown in Figures 2a,b and 3b,f. In all cases, the spoiling moment (I) has been applied.

For W1 the standard single‐frequency EPG approach is in excellent agreement with the measured flip angles with an RMSE of 0.7°. The same approach, however, fails to generate an asymmetric spoiling curve and thus mismatches the measured spoiling curve in PVP5 (blue line and orange dots in Figure 8b).

Instead, by extending the EPG simulation and including interference effects in a two‐frequency model with fitting parameters of *w*/*φ* = 0.136/−0.18*π* (cf. Equation (3)), this leads to good agreement (RMSE = 2.2°) of the simulated and measured spoiling curves (orange curve and dots in Figure 8b). The frequency difference was set to Δ*ν* = 320 Hz to simulate the influence of the upfield‐shifted band in the ^1^H‐NMR spectra at 3 T.

At 7 T, the two‐frequency simulation model achieves agreement between measured and simulated results for both *TE* = 1.9 ms and *TE* = 3.0 ms shown in Figure 8c,d. For *TE* = 1.9 ms (Figure 8c), the optimized fitting parameters (*w*/*φ* = 0.052/0.21*π*) lead to an asymmetric spoiling curve with its minimum located at Φ_0_ = 60°, matching well the measured spoiling curve (Figure 8c) with an RMSE = 1.5°. As shown before, changing the *TE* alters the asymmetry of the measured curve with a minimum shifted to Φ_0_ = 120°, which is matched by the two‐frequency EPG simulations with an RMSE = 1.72° with fitting parameter values of *w*/*φ* = 0.078/−0.59*π*.

## 4 Discussion

In this work, unexpected errors in AFI‐based FA maps measured in PVP phantoms were reported and investigated at 3 T and 7 T. Initial images (Figure 1) revealed a dependency of the FA not only on Φ_0_ but also on the PVP concentration, which triggered the investigation of this effect.

Prior work showed [21, 22] that the accuracy of AFI measurements depends on the degree of applied gradient spoiling and on the applied RF spoiling phase increment Φ_0_. Consequently, acquisitions and simulations of spoiling curves have been performed by Nehrke [21] and Yarnykh [22] to quantify the impact of spoiling related errors on AFI and to provide recommendations for the choice of Φ_0_. While a general value of Φ_0_ = 129.3° was suggested by Nehrke, excluding materials with insufficient diffusion damping [21], Yarnykh proposed (1) Φ_0_ = 39° together with high gradient spoiling moments *A*_G1_/*A*_G2_ = 450/2250 mT ms/m for high field applications and (2) Φ_0_ = 34° for $B_{1}^{+}$ nonuniformities <50% with *A*_G1_/*A*_G2_ = 280/1400 mT ms/m [22].

In contrast to previous reports showing symmetric spoiling curves, our measurements on PVP/water mixtures (tube PVP1–8) revealed spoiling curves with asymmetric shapes (Figure 2) that had not been reported before. In addition, the measurements reveal for some *TE* values strong FA deviations (depending on the applied gradient spoiling moment), even for recommended Φ_0_ values. On the other hand, using solutions of water and contrast agent (W1–4) with matched *T*_1_ values showed the expected symmetric spoiling curves, thus confirming the published results [21, 22]. Thus, poor RF or gradient spoiling alone does not explain the errors in AFI measurements in PVP/water mixtures. Furthermore, spoiling curves should not depend on TE; however, this effect was observed for the PVP tubes (Figure 3).

A possible source of these errors and unexpected results is revealed by measurements of the *TE* dependency of the AFI signals (Figures 5 and 6) and ^1^H‐NMR spectroscopy (Figure 7). Theoretically, the signal strength of the GRE images used to calculate the AFI should decay exponentially with *TE*. This is true for the water tubes; however, all PVP experiments at 3 T (e.g., Figure 5b,d) show an oscillating signal curve. This effect can be caused by multiple different magnetization components in the voxel having different precession frequencies, which yields a beating phenomenon of the signal. Oscillations are also found at 7 T (Figure 6d) but with a different shape, possibly due to the altered precession frequency, and without a clear period.

The existence of off‐resonant signals is confirmed by the ^1^H‐NMR spectra which agree with spectra found in literature [28]. The fact that the residual water peak amplitude is an order of magnitude higher than the PVP peaks in the spectra appears contradictory to the strong signal oscillations found in Figure 5 (74% change in signal magnitude). The AFI, however, is acquired in a steady state and the *T*_1_ of the PVP peaks (*T*_1,20%PVP_ = 100 ms, *TR*_1_/*TR*_2_ = 25/125 ms) are short in comparison to *T*_1_ of water (*T*_1,20%PVP_ = 4000 ms). Thus, the water peak is stronger saturated as compared with the PVP peaks. The acquired spectra also explain the dependence of the measurement errors on the PVP ratio, which is illustrated in Figure 1. Increased PVP ratios broaden the PVP resonances, thus reducing their $T_{2}^{*}$ values to values comparable to typical *TE* values used in AFI ($T_{2}^{*}=5.7/1.6\;\mathrm{ms}$ for the 20/50 wt% PVP sample). This reduces their amplitude and their influence on the GRE signals measured during AFI acquisition.

Using standard EPG simulations without off‐resonant components matches well with the spoiling curve of W1 in Figure 8a and is comparable to results using Bloch‐Simulations [22] or configuration theory [21]. The same approach, however, fails to explain the asymmetric PVP spoiling curves (Figure 8b), indicating an additional effect not covered by the simulation. The observed *TE* dependence of the FA in PVP indicates the presence of at least one additional frequency component. The ^1^H‐NMR spectra show three relevant bands that could lead to signal interferences during image acquisition. However, to reduce the degrees of freedom of the simulation, only a two‐frequency model was realized (Equation (3)). Although a simplification, the resulting EPG‐simulated spoiling curves (Figure 8b–d) are in good agreement with the measured data. These findings support the hypothesis that signal interferences cause the errors observed in AFI of PVP solutions.

The PVP signal components have such a short $T_{2}^{*}$ that they can be assumed to be perfectly spoiled before the next TR and thus not influenced by gradient or RF spoiling. However, since the water signal component depends on the spoiling the TE‐dependence of the signal interference is altered by gradient or RF‐spoiling. Thus, even in the case of very strong or complete spoiling, signal interferences will occur and lead to FA deviations, as illustrated in Figure 4 showing the impact of both parameters, *TE* and gradient spoiling on the measured FA.

Underestimations of the flip angle are particularly severe in the context of coil validation measurements. Here, a high PVP ratio (≈50 wt%) is beneficial, since it reduces the influence of the off‐resonant PVP resonances due to their shortened $T_{2}^{*}$ times. However, the simulation of tissues with higher *ε*_*r*_ like heart or muscle tissue requires lower PVP ratios [11] in which case the shown effect becomes more important. Using long *TE* values for image acquisition further exploits the short $T_{2}^{*}$ times but also reduces image SNR. At 7 T, echo times longer than 3 ms are preferable while at 3 T, echo times above 4.5 ms or alternatively in the range of 2.1–3.5 ms minimize the influence of the interference‐based error mechanism. These recommendations apply for Φ_0_ = 120°. Furthermore, gradient spoiling moments should be maximized to achieve a high degree of spoiling as spoiling‐ and interference‐based errors add up in a non‐linear fashion as shown in Figure 4b. No general recommendations for Φ_0_ can be made as the spoiling curves depend on *TE* in the presence of interference‐based errors. If, however, their influence is minimized by the proposed actions, the spoiling recommendations proposed by Yarnykh [22] should be applicable. In the context of this static phantom setup, it should be acknowledged that other $B_{1}^{+}$ mapping methods are also available for coil validation measurements like Bloch‐Siegert shift $B_{1}^{+}$ mapping [29] or magnetic resonance fingerprinting‐based methods [26].

Although this work is focused on PVP‐based phantom fillings, the interference effect described here is in principle not limited to PVP and might also appear in other phantom mixtures. As a general test for the existence of interference‐based errors in a substance, we suggest measuring the *TE* dependency of the AFI. Oscillatory behavior of the resulting curve (Figure 6) is an indication that the described interference effects are present in the material.

## 5 Conclusion

AFI in PVP‐based phantom fillings shows substantial deviations from the reference flip angle at 3 T and 7 T that cannot be explained by incomplete spoiling conditions alone. Multiple interfering signal components with different precession frequencies inside one voxel must be considered to explain the effect. Its presence can be identified by a *TE* dependence of the AFI. Errors caused by the interference‐based error mechanism are reduced by increasing *TE* values and by applying high gradient spoiling moments. Knowledge of this effect prevents unexpected errors in AFI‐based $B_{1}^{+}$ measurements. This will benefit AFI applications like UHF‐coil validation measurements, EPT and the correction of *T*_1_ maps.

## Supporting information


**Figure S1:** Spoiling curves of tube W2 and PVP6 with matching *T*_1_ acquired at 3 T with *TE* = 1.9 ms and spoiling gradient moments of *A*_G1_/*A*_G2_ = 117.5/587.5 mT ms/m (a + b), *A*_G1_/*A*_G2_ = 234.9/1174.5 mT ms/m (c + d), and *A*_G1_/*A*_G2_ = 469.7/2348.5 mT ms/m (e + f). The reference value for each measurement is indicated by a dashed line. All subfigures share the *x*‐ and *y*‐axis and error bars indicate the standard deviation in the tube.
**Figure S2:** Spoiling curves of tube W3 and PVP7 with matching *T*_1_ acquired at 3 T with *TE* = 1.9 ms and spoiling gradient moments of *A*_G1_/*A*_G2_ = 117.5/587.5 mT ms/m (a + b), *A*_G1_/*A*_G2_ = 234.9/1174.5 mT ms/m (c + d), and *A*_G1_/*A*_G2_ = 469.7/2348.5 mT ms/m (e + f). The reference value for each measurement is indicated by a dashed line. All subfigures share the *x*‐ and *y*‐axis and error bars indicate the standard deviation in the tube.
**Figure S3:** Spoiling curves of tube W4 and PVP8 with matching *T*_1_ acquired at 3 T with *TE* = 1.9 ms and spoiling gradient moments of *A*_G1_/*A*_G2_ = 117.5/587.5 mT ms/m (a + b), *A*_G1_/*A*_G2_ = 234.9/1174.5 mT ms/m (c + d), and *A*_G1_/*A*_G2_ = 469.7/2348.5 mT ms/m (e + f). The reference value for each measurement is indicated by a dashed line. All subfigures share the *x*‐ and *y*‐axis and error bars indicate the standard deviation in the tube.
**Figure S4:** Spoiling curves of tube PVP1 and PVP2 acquired at 3 T with *TE* = 1.9 ms and spoiling gradient moments of *A*_G1_/*A*_G2_ = 117.5/587.5 mT ms/m (a + b), *A*_G1_/*A*_G2_ = 234.9/1174.5 mT ms/m (c + d), and *A*_G1_/*A*_G2_ = 469.7/2348.5 mT ms/m (e + f). The reference value for each measurement is indicated by a dashed line. All subfigures share the *x*‐ and *y*‐axis and error bars indicate the standard deviation in the tube.
**Figure S5:** Spoiling curves of tube PVP3 and PVP4 acquired at 3 T with *TE* = 1.9 ms and spoiling gradient moments of *A*_G1_/*A*_G2_ = 117.5/587.5 mT ms/m (a + b), *A*_G1_/*A*_G2_ = 234.9/1174.5 mT ms/m (c + d) and *A*_G1_/*A*_G2_ = 469.7/2348.5 mT ms/m (e + f). The reference value for each measurement is indicated by a dashed line. All subfigures share the *x*‐ and *y*‐axis and error bars indicate the standard deviation in the tube.
**Figure S6:** Echo time dependency of the FA (a) and the signal strength in the GRE images (b) acquired in a phantom containing water and 5% gelatin. Φ_0_ was fixed to 120° and gradient spoiling moments set to *A*_G1_/*A*_G2_ = 117.4/587.0 mT ms/m. The oscillatory behavior of the signal curves found in PVP‐based phantom fillings is also visible here. The impact on the measured FA, however, is smaller compared with PVP.
**Figure S7:** FA maps of the phantom holder filled with six tubes acquired at 7 T with (a) the PEX method as a reference image and (b) by AFI (*A*_G1_/*A*_G2_ = 117.5/587.5 mT ms/m, *TE* = 1.9 ms, Φ_0_ = 40°). The effect of varying FA values in the AFI image is qualitatively less present than at 3 T. The reference voltage was set to achieve FAs in the working range of the AFI (20°–70°) inside the tubes. However, this led to an increase of FA over 90° in the center of the phantom holder. These values are underestimated by the AFI due to saturation effects that appear for FAs close to 90°.
**Figure S8:** FA maps of the phantom holder with two tubes containing 20 wt% PVP once with the preservative Germaben II (G) and once without it (NG). Both maps were acquired at 7 T with *A*_G1_/*A*_G2_ = 117.5/587.5 mT ms/m, *TE* = 1.9 ms and Φ_0_ = 60°. Exchanging the two tubes leads only to slight changes of the measured FA below 1.1° in the two ROIs (white circles with annotated mean FA). The influence of the preservative was therefore assumed to be negligible.
**Figure S9:** Simulated and measured spoiling curves for (a) W1 and (b) PVP5 at 3 T with *TE* = 1.9 ms and *A*_G1_/*A*_G2_ = 234.9/1174.5 mT ms/m, (c) W1 and (d) PVP5 at 3 T with *TE* = 1.9 ms and *A*_G1_/*A*_G2_ = 469.7/2348.5 mT ms/m as well as (e) W1 and (f) PVP5 at 7 T with *TE* = 2.5 ms and *A*_G1_/*A*_G2_ = 117.4/587.0 mT ms/m. Single‐frequency simulations match with the measured spoiling curves of W1 but do not show the asymmetric shape of the PVP curves. Adding an additional precession frequency to the simulation leads to good agreement of simulated and measured PVP spoiling curves. The measured spoiling curves without the simulation results are shown in Figures 2 and 3. The reference values used for the spoiling curve simulations are indicated by dashed lines and axes are shared between all subfigures.
