# Supplementary material for: Flip Angle Errors in Actual Flip Angle Imaging Using Polyvinylpyrrolidone/Water‐Based Phantoms
Source: Magn Reson Med. 2025 Oct 18;95(3):1489–502. doi: 10.1002/mrm.70136 (PMC12746354; doi:10.1002/mrm.70136)
Supplement: Supplementary file 1 — Figure S1: Spoiling curves of tube W2 and PVP6 with matching T 1 acquired at 3 T with TE = 1.9 ms and spoiling gradient moments of A G1/A G2 = 117.5/587.5 mT ms/m (a + b), A G1/A G2 = 234.9/1174.5 mT ms/m (c + d), and A G1/A G2 = 469.7/2348.5 mT ms/m (e + f). The reference value for each measurement is indicated by a dashed line. All subfigures share the x‐ and y‐axis and error bars indicate the standard deviation in the tube. Figure S2: Spoiling curves of tube W3 and PVP7 with matching T 1 acquired at 3 T with TE = 1.9 ms and spoiling gradient moments of A G1/A G2 = 117.5/587.5 mT ms/m (a + b), A G1/A G2 = 234.9/1174.5 mT ms/m (c + d), and A G1/A G2 = 469.7/2348.5 mT ms/m (e + f). The reference value for each measurement is indicated by a dashed line. All subfigures share the x‐ and y‐axis and error bars indicate the standard deviation in the tube. Figure S3: Spoiling curves of tube W4 and PVP8 with matching T 1 acquired at 3 T with TE = 1.9 ms and spoiling gradient moments of A G1/A G2 = 117.5/587.5 mT ms/m (a + b), A G1/A G2 = 234.9/1174.5 mT ms/m (c + d), and A G1/A G2 = 469.7/2348.5 mT ms/m (e + f). The reference value for each measurement is indicated by a dashed line. All subfigures share the x‐ and y‐axis and error bars indicate the standard deviation in the tube. Figure S4: Spoiling curves of tube PVP1 and PVP2 acquired at 3 T with TE = 1.9 ms and spoiling gradient moments of A G1/A G2 = 117.5/587.5 mT ms/m (a + b), A G1/A G2 = 234.9/1174.5 mT ms/m (c + d), and A G1/A G2 = 469.7/2348.5 mT ms/m (e + f). The reference value for each measurement is indicated by a dashed line. All subfigures share the x‐ and y‐axis and error bars indicate the standard deviation in the tube. Figure S5: Spoiling curves of tube PVP3 and PVP4 acquired at 3 T with TE = 1.9 ms and spoiling gradient moments of A G1/A G2 = 117.5/587.5 mT ms/m (a + b), A G1/A G2 = 234.9/1174.5 mT ms/m (c + d) and A G1/A G2 = 469.7/2348.5 mT ms/m (e + f). The reference value for each measurement is indic [file MRM-95-1489-s001.docx]

# Supporting Information


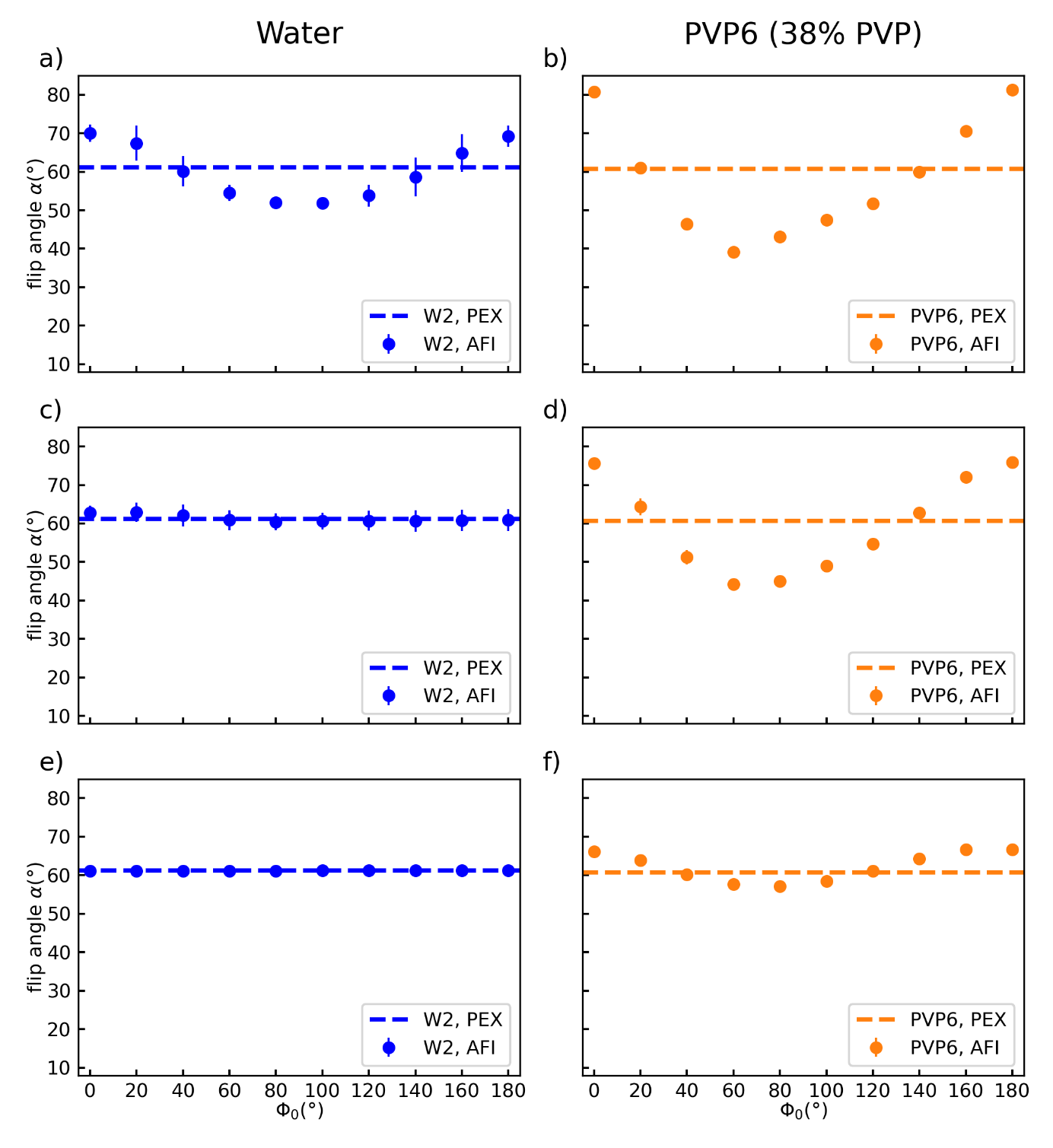


*Supporting Information Figure S1: Spoiling curves of tube W2 and PVP6 with matching T_1_ acquired at 3T with TE=1.9ms and spoiling gradient moments of A_G1_/A_G2_=117.5/587.5 mT·ms/m (a+b), A_G1_/A_G2_= 234.9/1174.5 mT·ms/m (c+d) and A_G1_/A_G2_=469.7/2348.5 mT·ms/m (e+f). The reference value for each measurement is indicated by a dashed line. All subfigures share the x- and y-axis and error bars indicate the standard deviation in the tube.*


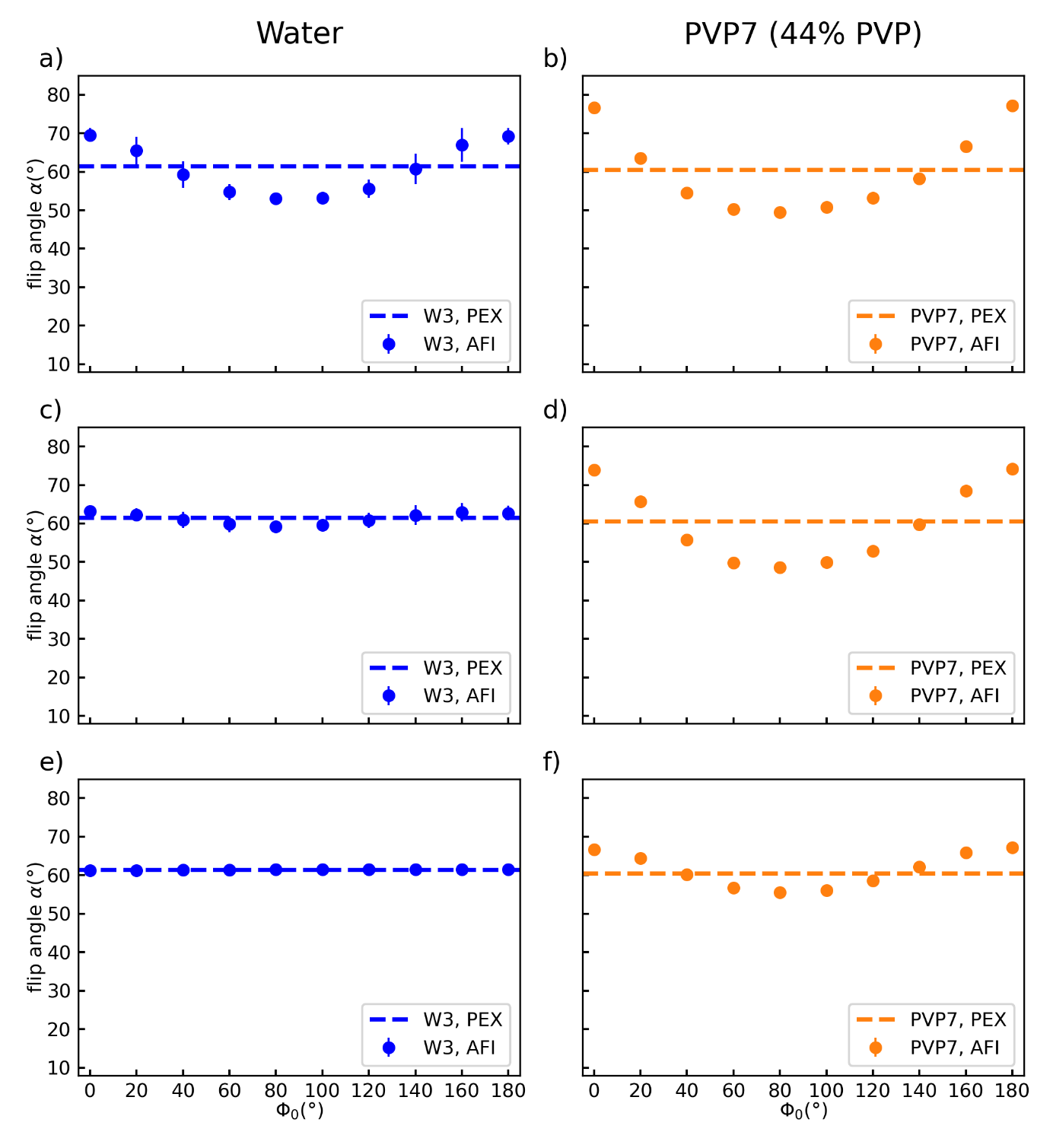
*Supporting Information Figure S2: Spoiling curves of tube W3 and PVP7 with matching T_1_ acquired at 3T with TE=1.9ms and spoiling gradient moments of A_G1_/A_G2_=117.5/587.5 mT·ms/m (a+b), A_G1_/A_G2_= 234.9/1174.5 mT·ms/m (c+d) and A_G1_/A_G2_=469.7/2348.5 mT·ms/m (e+f). The reference value for each measurement is indicated by a dashed line. All subfigures share the x- and y-axis and error bars indicate the standard deviation in the tube.*


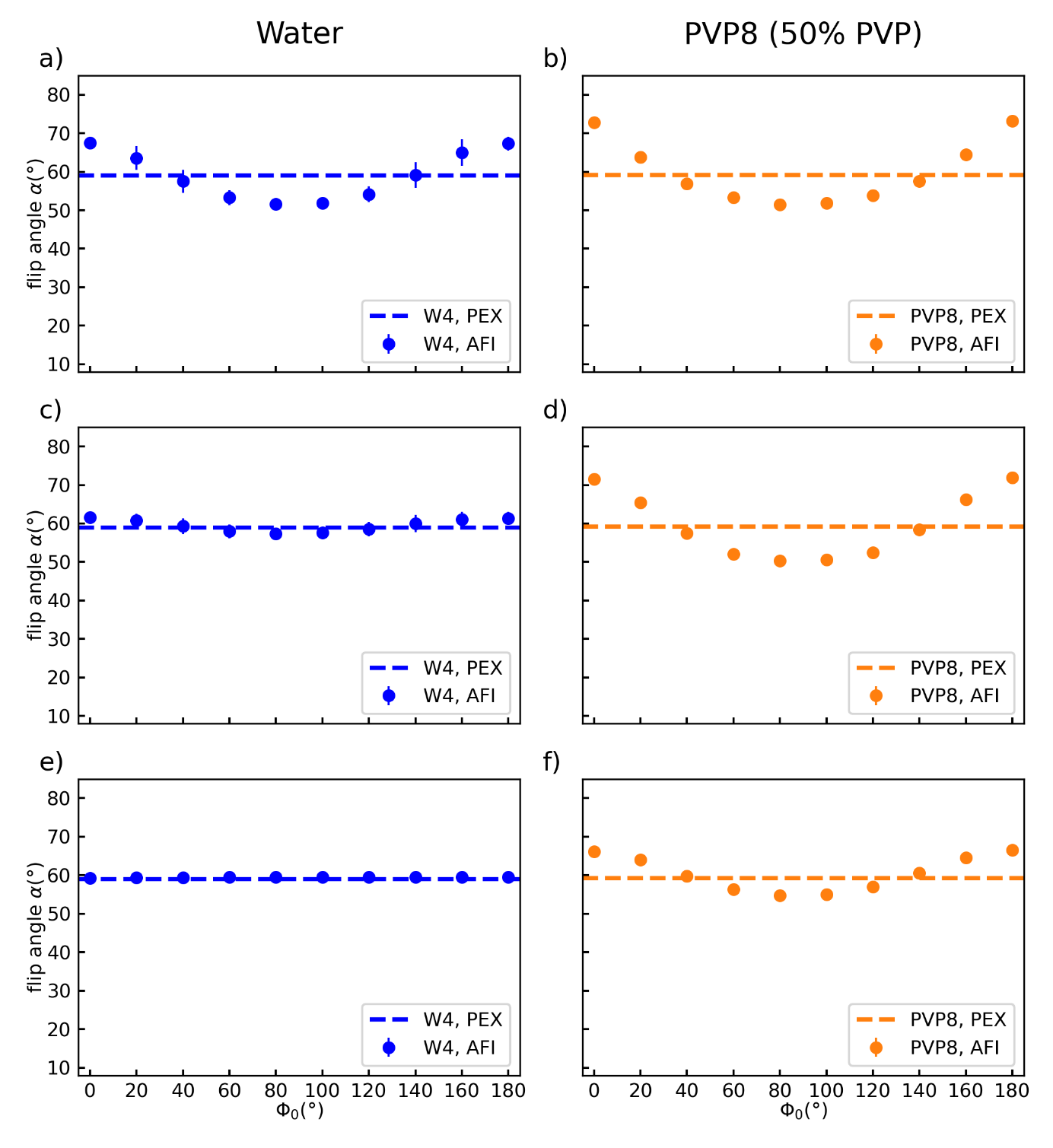
*Supporting Information Figure S3: Spoiling curves of tube W4 and PVP8 with matching T_1_ acquired at 3T with TE=1.9ms and spoiling gradient moments of A_G1_/A_G2_=117.5/587.5 mT·ms/m (a+b), A_G1_/A_G2_= 234.9/1174.5 mT·ms/m (c+d) and A_G1_/A_G2_=469.7/2348.5 mT·ms/m (e+f). The reference value for each measurement is indicated by a dashed line. All subfigures share the x- and y-axis and error bars indicate the standard deviation in the tube.*


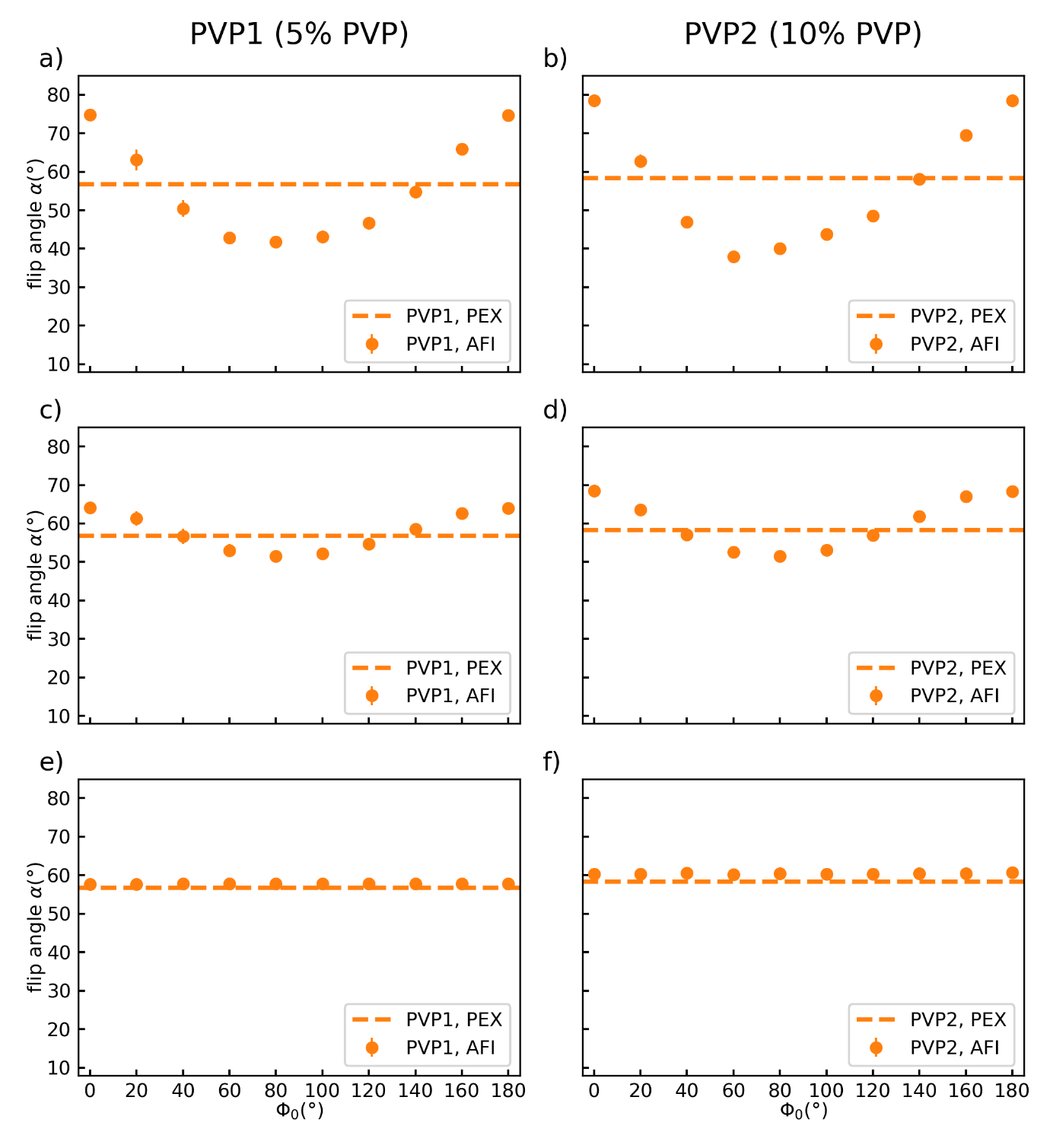
*Supporting Information Figure S4: Spoiling curves of tube PVP1 and PVP2 acquired at 3T with TE=1.9ms and spoiling gradient moments of A_G1_/A_G2_=117.5/587.5 mT·ms/m (a+b), A_G1_/A_G2_= 234.9/1174.5 mT·ms/m (c+d) and A_G1_/A_G2_=469.7/2348.5 mT·ms/m (e+f). The reference value for each measurement is indicated by a dashed line. All subfigures share the x- and y-axis and error bars indicate the standard deviation in the tube.*


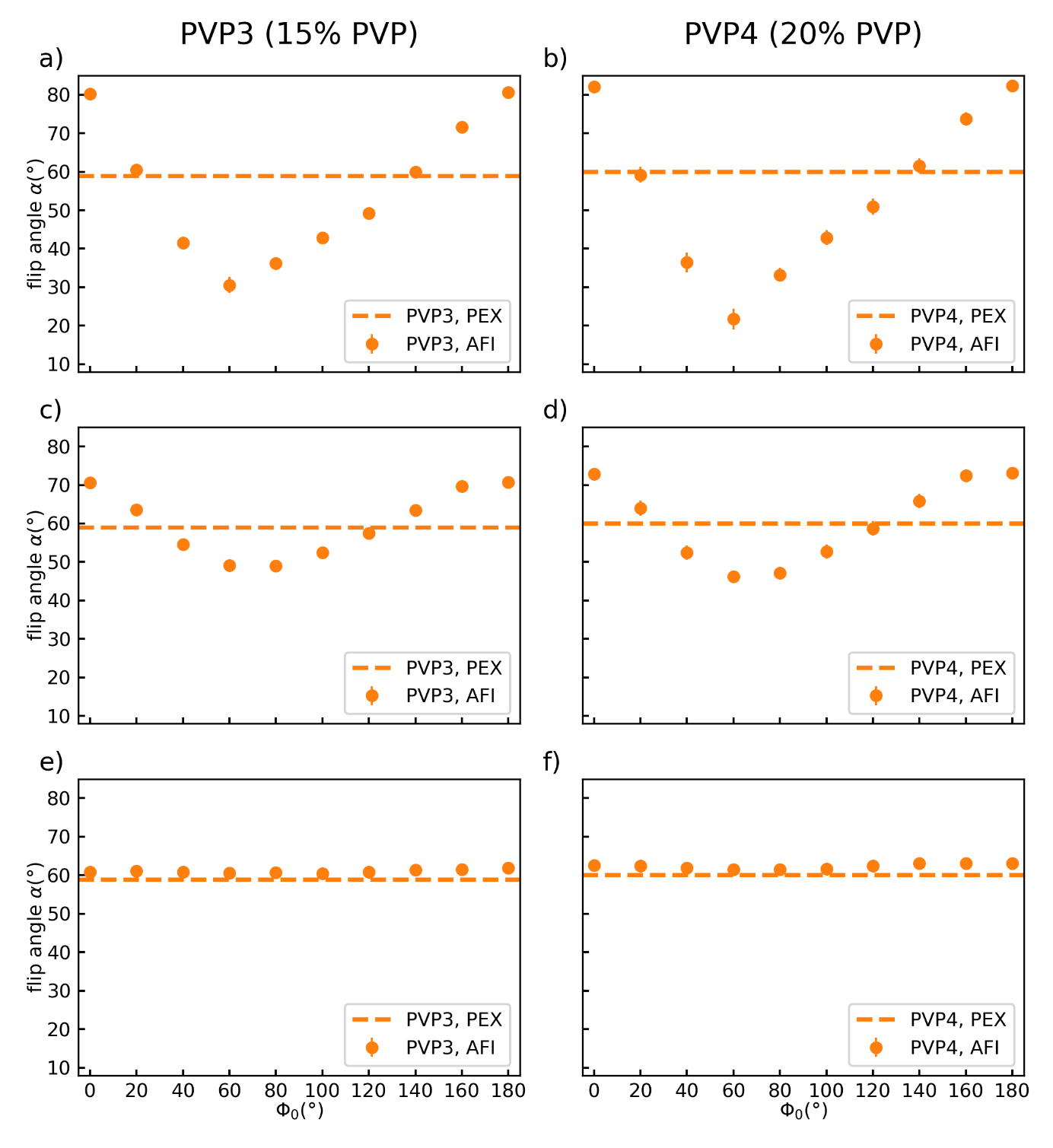
*Supporting Information Figure S5: Spoiling curves of tube PVP3 and PVP4 acquired at 3T with TE=1.9ms and spoiling gradient moments of A_G1_/A_G2_=117.5/587.5 mT·ms/m (a+b), A_G1_/A_G2_= 234.9/1174.5 mT·ms/m (c+d) and A_G1_/A_G2_=469.7/2348.5 mT·ms/m (e+f). The reference value for each measurement is indicated by a dashed line. All subfigures share the x- and y-axis and error bars indicate the standard deviation in the tube.*


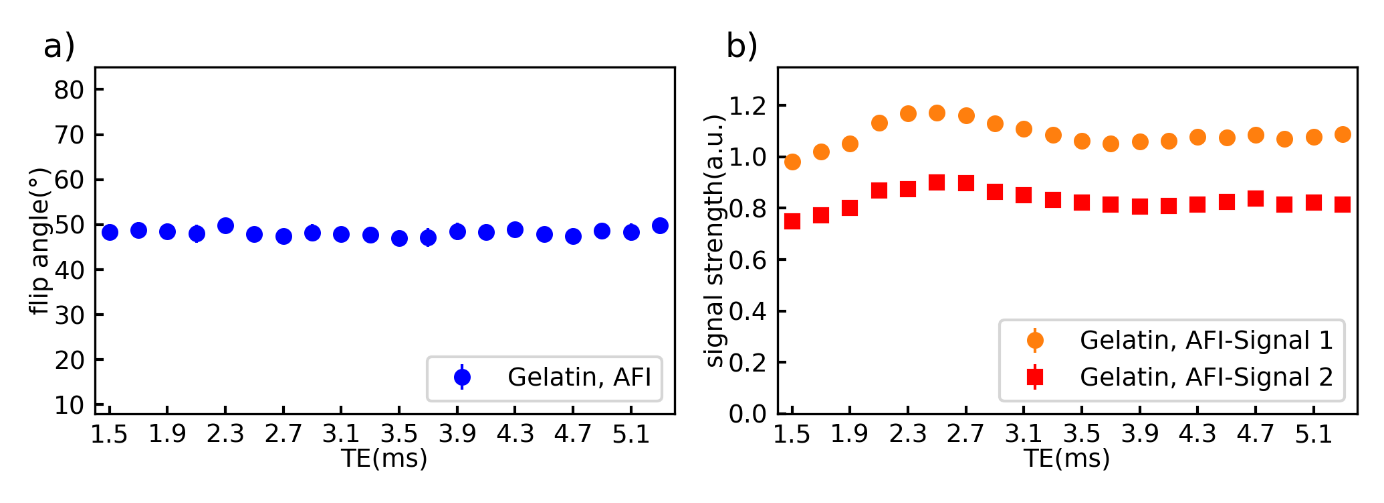


*Supporting Information Figure S6: Echo time dependency of the FA (a) and the signal strength in the GRE images (b) acquired in a phantom containing water and 5% gelatin. Φ_0_ was fixed to 120° and gradient spoiling moments set to A_G1_/A_G2_=117.4/587.0 mT·ms/m. The oscillatory behavior of the signal curves found in PVP-based phantom fillings is also visible here. The impact on the measured FA, however, is smaller compared to PVP.*


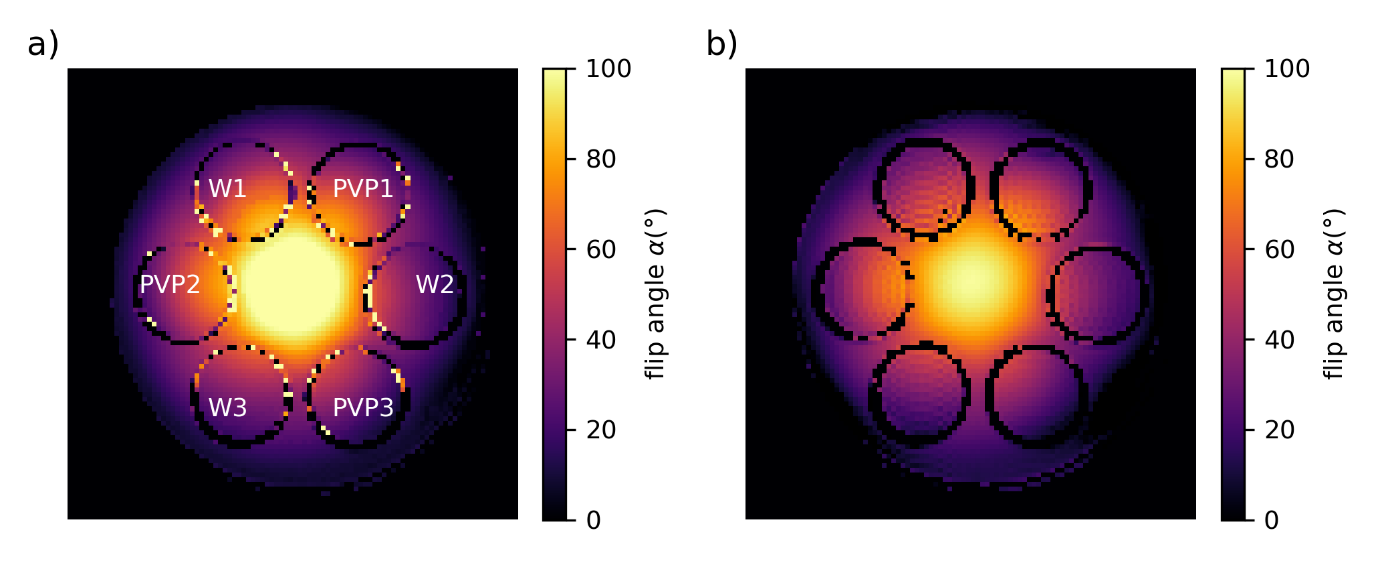
 *Supporting Information Figure S7: FA maps of the phantom holder filled with six tubes acquired at 7T with a) the PEX method as a reference image and b) by AFI (A_G1_/A_G2_=117.5/587.5 mT·ms/m, TE=1.9ms, Φ_0_=40°). The effect of varying FA values in the AFI image is qualitatively less present than at 3T. The reference voltage was set to achieve FAs in the working range of the AFI (20°-70°) inside the tubes. However, this led to an increase of FA over 90° in the center of the phantom holder. These values are underestimated by the AFI due to saturation effects that appear for FAs close to 90°.*


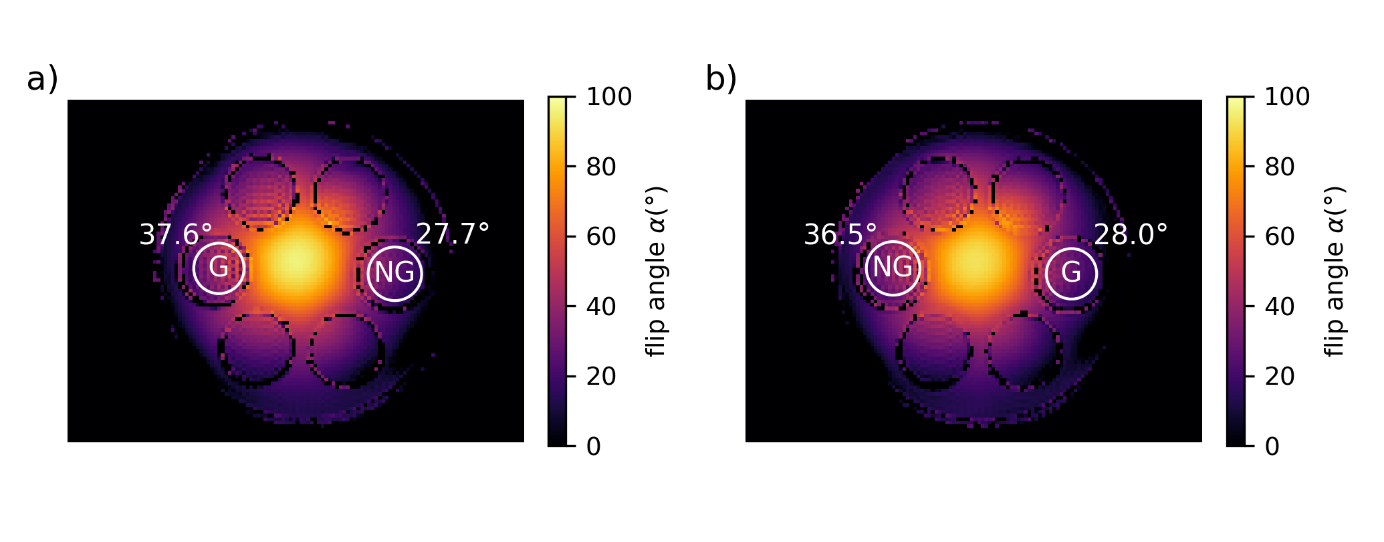
*Supporting Information Figure S8: FA maps of the phantom holder with two tubes containing 20wt.% PVP once with the preservative Germaben II (G) and once without it (NG). Both maps were acquired at 7T with A_G1_/A_G2_=117.5/587.5 mT·ms/m, TE=1.9ms and Φ_0_=60°. Exchanging the two tubes leads only to slight changes of the measured FA below 1.1° in the two ROIs (white circles with annotated mean FA). The influence of the preservative was therefore assumed to be negligible.*


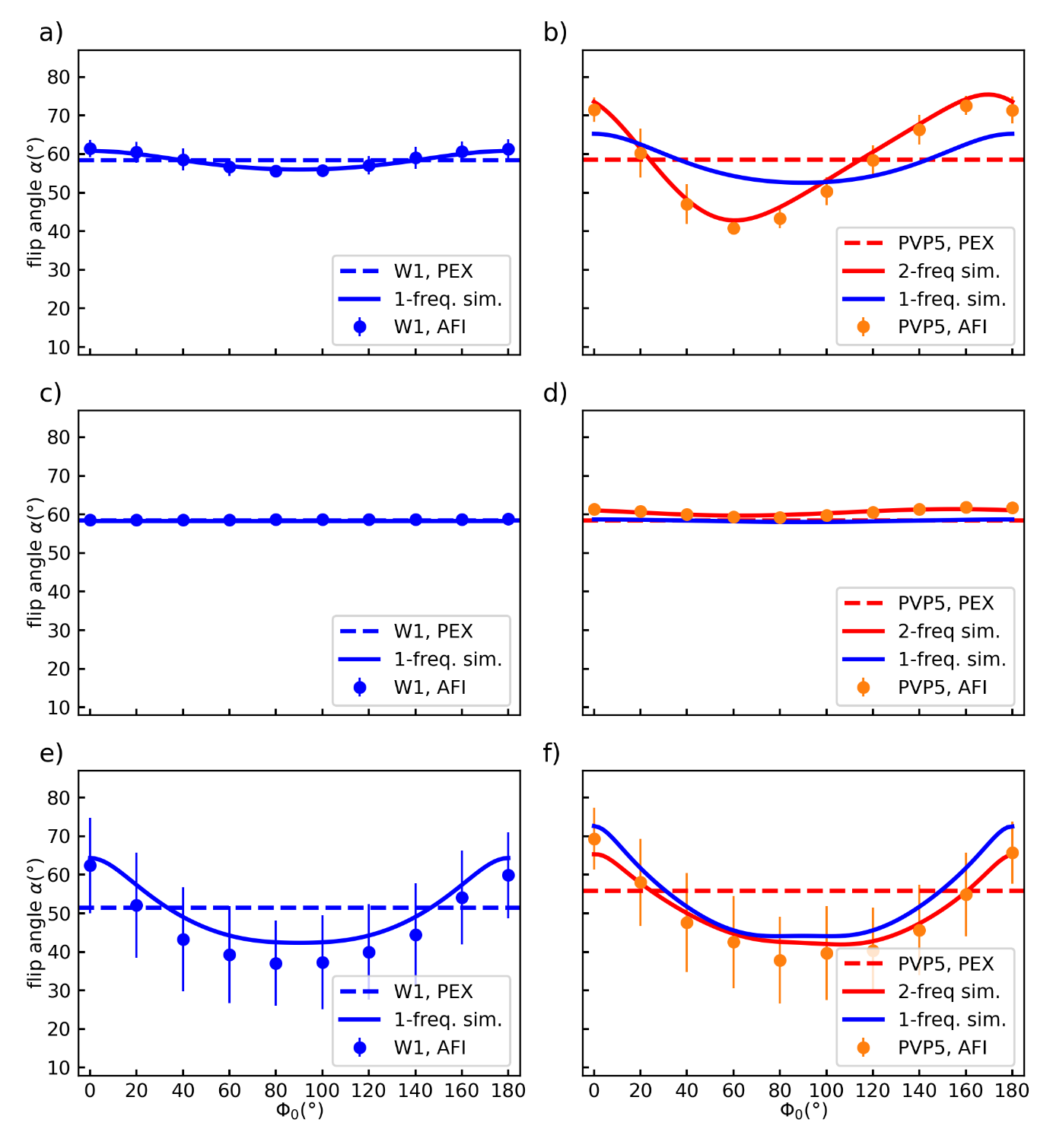
*Supporting Information Figure S9: Simulated and measured spoiling curves for a) W1 and b) PVP5 at 3T with TE=1.9ms and A_G1_/A_G2_=234.9/1174.5 mT·ms/m, c) W1 and d) PVP5 at 3T with TE=1.9ms and A_G1_/A_G2_=469.7/2348.5 mT·ms/m as well as e) W1 and f) PVP5 at 7T with TE=2.5ms and A_G1_/A_G2_=117.4/587.0 mT·ms/m. Single-frequency simulations match with the measured spoiling curves of W1 but do not show the asymmetric shape of the PVP curves. Adding an additional precession frequency to the simulation leads to good agreement of simulated and measured PVP spoiling curves. The measured spoiling curves without the simulation results are shown in Fig.2+3. The reference values used for the spoiling curve simulations are indicated by dashed lines and axes are shared between all subfigures.*
